# *Staphylococcus aureus* Membrane-Derived Vesicles Promote Bacterial Virulence and Confer Protective Immunity in Murine Infection Models

**DOI:** 10.3389/fmicb.2018.00262

**Published:** 2018-02-20

**Authors:** Fatemeh Askarian, John D. Lapek, Mitesh Dongre, Chih-Ming Tsai, Monika Kumaraswamy, Armin Kousha, J. Andrés Valderrama, Judith A. Ludviksen, Jorunn P. Cavanagh, Satoshi Uchiyama, Tom E. Mollnes, David J. Gonzalez, Sun N. Wai, Victor Nizet, Mona Johannessen

**Affiliations:** ^1^Research Group of Host Microbe Interactions, Department of Medical Biology, Faculty of Health Sciences, UiT – The Arctic University of Norway, Tromsø, Norway; ^2^Division of Host-Microbe Systems and Therapeutics, Department of Pediatrics, University of California, San Diego, La Jolla, CA, United States; ^3^Department of Pharmacology, University of California, San Diego, La Jolla, CA, United States; ^4^The Laboratory for Molecular Infection Medicine Sweden, Department of Molecular Biology, Umeå University, Umeå, Sweden; ^5^Division of Infectious Diseases, Department of Medicine, University of California, San Diego, La Jolla, CA, United States; ^6^Research Laboratory, Nordland Hospital, Bodø, Norway; ^7^Department of Pediatrics and Neonatal Medicine, University Hospital of North Norway, University Hospital of North Norway, Tromsø, Norway; ^8^Department of Pediatrics, Faculty of Health Sciences, UiT – The Arctic University of Norway, Tromsø, Norway; ^9^Faculty of Health Sciences, K. G. Jebsen Thrombosis Research and Expertise Center, UiT – The Arctic University of Norway, Tromsø, Norway; ^10^Department of Immunology, Oslo University Hospital, University of Oslo, Oslo, Norway; ^11^K.G. Jebsen Inflammation Research Centre, University of Oslo, Oslo, Norway; ^12^Center for Molecular Inflammation Research, Norwegian University of Science and Technology, Trondheim, Norway; ^13^Skaggs School of Pharmacy and Pharmaceutical Sciences, University of California, San Diego, La Jolla, CA, United States

**Keywords:** *Staphylococcus aureus*, membrane-derived vesicles, proteomics, systemic infection, protective immunity

## Abstract

*Staphylococcus aureus* produces membrane-derived vesicles (MVs), which share functional properties to outer membrane vesicles. Atomic force microscopy revealed that *S. aureus*-derived MVs are associated with the bacterial surface or released into the surrounding environment depending on bacterial growth conditions. By using a comparative proteomic approach, a total of 131 and 617 proteins were identified in MVs isolated from *S. aureus* grown in Luria-Bertani and brain-heart infusion broth, respectively. Purified *S. aureus* MVs derived from the bacteria grown in either media induced comparable levels of cytotoxicity and neutrophil-activation. Administration of exogenous MVs increased the resistance of *S. aureus* to killing by whole blood or purified human neutrophils *ex vivo* and increased *S. aureus* survival *in vivo*. Finally, immunization of mice with *S. aureus*-derived MVs induced production of IgM, total IgG, IgG1, IgG2a, and IgG2b resulting in protection against subcutaneous and systemic *S. aureus* infection. Collectively, our results suggest *S. aureus* MVs can influence bacterial–host interactions during systemic infections and provide protective immunity in murine models of infection.

## Introduction

Many pathogenic Gram-negative bacteria species extend their virulence potential by releasing spherical buds, derived from the outer membrane, so-called outer membrane vesicles (OMVs; reviewed in Bomberger et al., 2009; Kulp and Kuehn, 2010; Kaparakis-Liaskos and Ferrero, 2015; Yáñez-Mó et al., 2015). OMVs play an essential role in the long-distance delivery of intact bacterial effectors (Kuehn and Kesty, 2005; Mashburn and Whiteley, 2005; Schooling and Beveridge, 2006; Ellis and Kuehn, 2010; Kulp and Kuehn, 2010; Gurung et al., 2011; Jun et al., 2013; Nho et al., 2015; Jeon et al., 2016) by protecting the cargo against the harsh extracellular environment (reviewed in Bomberger et al., 2009; Kulp and Kuehn, 2010; Kaparakis-Liaskos and Ferrero, 2015; Schwechheimer and Kuehn, 2015; Yáñez-Mó et al., 2015). The release of OMVs can benefit the microbe, mediating key microbial interactions with the human host and within bacterial communities (reviewed in Bomberger et al., 2009; Kulp and Kuehn, 2010; Kaparakis-Liaskos and Ferrero, 2015; Yáñez-Mó et al., 2015). Diverse functions ascribed to OMVs, even in the absence of live bacterial cells, include promotion of virulence, biofilm formation, signal transduction, cytotoxicity, and host pathology (Kuehn and Kesty, 2005; Mashburn and Whiteley, 2005; Schooling and Beveridge, 2006; Ellis and Kuehn, 2010; Kulp and Kuehn, 2010; Gurung et al., 2011; Jun et al., 2013; Brown et al., 2015; Nho et al., 2015; Jeon et al., 2016). Furthermore, OMVs contain various pathogen-associated molecular patterns, which can modulate the host pro- and/or anti-inflammatory responses (reviewed in Kuehn and Kesty, 2005; Kaparakis-Liaskos and Ferrero, 2015; Schwechheimer and Kuehn, 2015) and subsequently influence recruitment of immune cells to sites of inflammation (reviewed in Lee, 2012; Kaparakis-Liaskos and Ferrero, 2015; Schwechheimer and Kuehn, 2015).

In recent years, several Gram-positive bacteria and mycobacteria such as *Staphylococcus aureus, Mycobacterium ulcerans, Mycobacterium tuberculosis, Mycobacterium bovis bacilli, Bacillus* spp., *Streptococcus* spp., *Clostridium perfringens*, and *Listeria monocytogenes* have been found to produce membrane-derived vesicles (MVs) during *in vitro* culture and/or *in vivo* murine infection (Marsollier et al., 2007; Lee et al., 2009; Lee J.H. et al., 2013; Rivera et al., 2010; Gurung et al., 2011; Hong et al., 2011; Prados-Rosales et al., 2011; Thay et al., 2013; Jiang et al., 2014; Haas and Grenier, 2015; Resch et al., 2016). Thus, vesicle generation may be considered a ubiquitous conserved secretory pathway among bacteria (Gurung et al., 2011; Lee, 2012). In Gram-negative bacteria, a phospholipid transporter was found to be associated with OMV formation (Roier et al., 2016). For Gram-positive bacteria it has been hypothesized that the membrane and luminal contents of MVs are derived from the cytoplasmic membrane and cytoplasm, respectively (Kato et al., 2002; MacDonald and Kuehn, 2012; Yáñez-Mó et al., 2015). Recently, a prophage-triggered mechanism noted to endolysin expression and consequently peptidoglycan damage was identified as a putative mechanism for MV formation in the Gram-positive model organism, *Bacillus subtilis* (Toyofuku et al., 2017). However, the exact mechanism of MV biogenesis and transport through the thick cell wall of Gram-positive bacteria remains unknown.

*Staphylococcus aureus* can cause a wide array of invasive human diseases due to its ability to disarm the innate immune system with an extensive armamentarium of immune evasion factors (Foster et al., 2014). The pathogen is the most common cause of hospital-acquired infections, while community-acquired *S. aureus* infections are rapidly emerging as a public health problem (reviewed in Foster, 2009). Previous *in vitro* studies indicate that MVs play a critical role in the delivery of *S. aureus* toxins into host cells via interactions with the cholesterol-rich micro-domains of the host cell plasma membranes, whereupon host cell death may ensue (Gurung et al., 2011; Thay et al., 2013). Besides, *S. aureus*-derived MVs produce biofilm formation (He et al., 2017) and harbor biologically active β-lactamases, thwarting antibiotic effectiveness extracellularly (Lee J. et al., 2013). The potential role for *S. aureus* MVs in provoking atopic dermatitis-like skin (Hong et al., 2011; Jun et al., 2017) and neutrophilic pulmonary (Kim et al., 2012) inflammation have also been described.

Previous studies have revealed *S. aureus*-derived MVs carry a complex arsenal of virulence factors (Lee et al., 2009; Gurung et al., 2011; Jeon et al., 2016). However, the potential role that MVs play in *S. aureus* pathogenesis remains incompletely or poorly characterized. Furthermore, whether bacterial growth conditions influence MV cargo, and downstream host responses to MV exposure, are not yet understood. In this study, we examined the interactions between exogenously derived *S. aureus* MVs with several immune processes. Moreover, the immunogenic properties of *S. aureus* MVs and their ability to confer protection in murine models of sepsis and skin infection were also explored.

## Materials and Methods

### Bacterial Strains and Mammalian Cell Lines

*Staphylococcus aureus* subsp. *aureus* Rosenbach MSSA476 was purchased from LGC standard AB (ATCC- BAA-1721, Sweden), while *S. aureus* USA300-MRSA (TCH1516, ATCC BAA-1717) and its isogenic mutant USA300-MRSAΔHla were provided by Prof. V. Nizet. Keratinocytes (HaCaT cells; Boukamp et al., 1988) and monocytes (THP-1 cells) were purchased from PromoCell (Germany) and ATCC (United States), respectively. HaCaT cells were maintained in Dulbecco’s modified Eagle’s medium (Sigma–Aldrich, Germany), supplemented with 10% (v/v) fetal bovine serum (FBS; Invitrogen Life Technologies, United States), penicillin (100 units/ml), and 100 μg/ml streptomycin (Sigma–Aldrich, Germany) in a CO_2_ incubator (5% CO_2_) at 37°C. THP-1 cells were maintained in RPMI 1640 medium with 2 mM L-glutamine (Gibco, Life Technologies, United Kingdom) supplemented with 10% (v/v) FBS, 4.5 g/l glucose (Sigma–Aldrich, Germany), 10 mM HEPES (Sigma–Aldrich, Germany), and 1.0 mM sodium pyruvate (Sigma–Aldrich, Germany) supplemented with 0.05 mM 2-mercaptoethanol (Sigma–Aldrich, Germany).

### Atomic Force Microscopy (AFM)

Atomic force microscopy (AFM) analysis of *S. aureus* MSSA476 cultivated on Luria-Bertani (LB) agar (LA), brain-heart infusion (BHI) agar, and blood agar were carried out as described previously (Thay et al., 2013). Briefly, bacterial cells were suspended in ultrapure water and placed on a freshly cleaved mica surface. The samples were incubated for approximately 5 min at room temperature and blotted dry before being placed into a desiccator. Representative images were collected by a Nanoscope V Atomic Force Microscope (Bruker AXS, Germany).

### Transmission Electron Microscopy (TEM)

*Staphylococcus aureus* MSSA476 was cultivated on LA plate. The bacterial pellet was carefully resuspended in 0.1 M phosphate buffer pH 7.4. Thereafter, 1.5 μl of the sample was applied to glow-discharged, 300 mesh, copper grids for 1.5 min and quickly washed in two drops of phosphate buffer. Bacterial cells were briefly fixed in 2% paraformaldehyde in phosphate buffer. Grids were blocked with 0.5% cold fish skin gelatin (Sigma, Stockholm, Sweden) diluted in phosphate buffer, incubated with anti-*Staphylococcus aureus* antibody (Abcam, ab20002), which specifically recognizes an epitope of peptidoglycan. The primary antibody was diluted in blocking solution (1:30), washed in phosphate buffer and incubated in blocking solution (1:25) with 10 nm protein A-gold (Cell Microscopy Core, Utrecht). Grids were washed in MQ water, briefly fixed with 2.5% glutaraldehyde and washed before they were negatively stained with 1.5% uranyl acetate (TAAB, Berks, United Kingdom), 2 × 15 s (vesicles) and 20 s (bacterial cells). Samples were examined with Talos 12°C (FEI, Eindhoven, Netherlands) operating at 120 kV. Micrographs were acquired with a Ceta 16M CCD camera (FEI, Eindhoven, Netherlands) using transmission electron microscopy (TEM) Image and Analysis software ver. 4.15 (FEI, Eindhoven, Netherlands).

The negative-stain TEM analysis on the purified MVs was performed as previously described (Resch et al., 2016), with minor modification. Briefly, 5–6 μl of purified MVs were placed on Formvar-coated, 75 mesh, copper grids for 5 min at room temperature. The sample was quickly washed with ultrapure water (4×, 1–2 min each). Negative staining was performed using 2% methylcellulose and 3% uranyl acetate for 2 min on ice. The excess stain was removed, and the sample was allowed to dry. Imaging was performed using JEOL JEM 1010 (JEOL, United States) operating at 80 kV. A grid treated for TEM visualization in the absence of MVs, served as negative control.

### Isolation, Fractionation, and Quantification of MVs

*Staphylococcus aureus* MSSA476, USA300 MRSA, and its isogenic mutant USA300 MRSAΔHla were grown overnight at 37°C in LB and/or BHI broth. MVs were isolated from the overnight culture (diluted 1:100 and grown for 12–14 h). A pilot study was performed to evaluate whether media choice influenced the viability of *S. aureus*. Analysis of *S. aureus* viability was performed using propidium iodide (PI) staining (Invitrogen Life Technologies, United States) and flow cytometry. *S. aureus* treated with gentamicin (100 μg/ml) and lysostaphin (40 μg/ml) for 2 h at 37°C with shaking was stained with PI, and served as a positive control (Supplementary Figure 1). MVs were isolated and purified as previously described (Kim et al., 2012; Thay et al., 2013; Choi et al., 2015), with minor modifications. Briefly, the culture supernatants were prepared by centrifuging the bacterial cultures at 6,000 × *g* for 20 min at 4°C using a JLA 10-500 rotor (Beckman Coulter, United States) and filtered through 0.22-μm vacuum-bottle top filters (Millipore, United States). Filtration was performed to remove bulk bacterial cells and cell debris (Choi et al., 2017). The bacteria-free supernatant was then centrifuged at 100,000 × *g* at 4°C for 3–4 h in either 45 or 50.2 Ti rotors (Beckman Instruments, Inc.). The MVs pellet was washed twice with PBS as described previously (Lee J. et al., 2013; Fulsundar et al., 2014; Choi et al., 2015; Resch et al., 2016), centrifuged at 100,000 × *g* at 4°C for an extra 3–4 h and finally re-suspended in PBS (Biochrom, Germany). The protein content of the isolated MVs was measured using a Direct Detector^TM^ (Millipore, United States). The sterility of the isolated MVs was examined by streaking small aliquots on blood/BHI agar plates followed by overnight incubation at 37°C.

In order to perform proteomic analysis, fractionation of MVs from *S. aureus* MSSA476 grown in LB and BHI broth was carried out by density gradient centrifugation using Optiprep (Sigma–Aldrich, Germany) as previously described (Elluri et al., 2014), with minor modifications. Briefly, different Optiprep/ultrapure water layers were sequentially added to the tube as follows: 400 μl (45%), 600 μl (35%), 600 μl (30%), 600 μl (25%), 600 μl (20%), 500 μl (15%), and 400 μl (10%). Finally, 400 μl of isolated MVs was loaded on top of the gradient followed by centrifugation at 180,000 × *g* for 3 h at 4°C using an SW60Ti rotor (Beckman Instruments Inc., United States). Thereafter, 200 μl aliquots were sequentially harvested and analyzed by SDS-PAGE followed by Coomassie Blue staining. Fractions showing the same protein profile on Coomassie gel were pooled, and the purity of the MVs was verified by negative-stain TEM and/or AFM imaging (examples are shown in Supplementary Figures 2, 3B). Finally, the amount of MVs was quantified using Pierce BCA Protein Assay Kit (Thermo Fisher Scientific, United States) or Direct Detector^TM^ according to the manufacturer’s instructions.

### Dynamic Light Scattering (DLS)

Particle size distribution of *S. aureus*-derived MVs was analyzed at 25°C using a Zetasizer Nano ZS particle analyzer (Malvern, United Kingdom). The purified MVs were diluted to a final concentration of approximately 10 μg/ml (protein) in PBS, and the size was estimated. The results are represented as the percentage of MVs with diameters measured in nanometers (nm).

### Lipid-Based Protein Immobilization (LPI) and In-Solution Processing

The purity of the recovered fractions of MVs after Optiprep was verified using AFM prior to further processing by Nanoxis Consulting AB^1^. For the proteomic analysis a digestion protocol using two different approaches including the lipid-based protein immobilization (LPI) Hexa Lane FlowCell (Karlsson et al., 2012) and an in-solution (In-sol) protocol were utilized. The LPI FlowCell system provides a novel platform specifically for characterization of the membrane proteins. Briefly, *S. aureus*-derived MVs were immobilized through membrane–gold interactions, on the flow cell surface. After 1 h incubation to ensure adhesion, the MVs were washed with PBS and further subjected to trypsin digestion of surface-exposed proteins in multiple steps. This process enhances sequence coverage, and the generated peptides can be further analyzed with mass spectrometry (MS) to identify the MV-associated proteins (Chooneea et al., 2010; Karlsson et al., 2012).

In the LPI method, samples were injected into three different LPI channels (40 μl in each channel) and immobilized on the channel-surfaces. The wash step was performed using a syringe pump by pumping through 400 μl (100 mM, pH 8.0) of tetraethylammonium bromide (TEAB) (Sigma–Aldrich, Germany). Next, 100 μl of trypsin solution (2 μg/ml of trypsin in ammonium bicarbonate, 20 mM, pH 8.0) were added to each channel. The samples were then treated with trypsin for 1 h to digest surface-associated proteins into peptides. For the In-sol approach, 80 μl of the MV samples were diluted in 40 μl PBS followed by the addition of glass beads (150–212 μm; Sigma–Aldrich, Germany). Thereafter, samples were processed with a bead-beating step prior to a 6 h In-sol digestion with 2 μg/ml of trypsin in ammonium bicarbonate (20 mM, pH 8.0) at a 1:1 (sample to trypsin) ratio. Next, the peptide samples were eluted with 200 μl of TEAB by a syringe pump (100 μl/min) and collected for MS analysis.

### Peptide Sample Preparation and Liquid Chromatography (LC) Gradient

The sample preparation was carried out by Nanoxis AB (see text footnote 1) as described previously (Karlsson et al., 2012), with minor modifications. PepClean C18 spin columns (Thermo Fisher Scientific, United States) were used to desalt the peptides according to the manufacturer’s instructions. The columns were dried and reconstituted using 3% HPLC grade acetonitrile (ACN) (Merck, Germany) supplemented with 0.1% formic acid (Sigma–Aldrich, Germany). The sample was injected on an Easy-nLC autosampler (Thermo Fisher Scientific, United States) and analyzed with an interfaced Q Exactive hybrid mass spectrometer (Thermo Fisher Scientific, United States). The peptides were trapped on a pre-column (45 mm × 0.075 mm i.d.) and separated on a reversed phase analytical column (200 mm × 0.075 mm) packed in-house with 3 μm Reprosil-Pur C18-AQ particles (Dr. Maisch, Ammerbuch, Germany). The nanoLC (liquid chromatography) gradient was run at 200 nl/min as follow: 7–27% ACN supplemented with 0.2% formic acid during 25 min, 27–40% ACN during 5 min, 40–80% during 5 min, and finally hold at 80% ACN for 10 min.

### Mass Spectrometry (MS) Settings

MS was performed by Nanoxis and run in a data-dependent positive ion mode. The ion spray voltage into the mass spectrometer and capillary temperature were adjusted to 1.8 kV and 320°C. Full scan (MS1) spectra were acquired in the Orbitrap over the *m/z* range 400–1,600, charge range 2–6 at a resolution of 70,000 until an AGC target value of 1e6 with a maximum injection time of 250 ms. MS/MS spectra were acquired using higher energy collision dissociation at 30% from *m/z* 110 for the 10 most abundant parent ions at a resolution of 35,000 using a precursor isolation window of 2 Da with an AGC target value of 100,000 and a maximum injection time of 110 ms. Dynamic exclusion during 30 s after selection for MS/MS was enabled to allow for detection of as many precursors as possible.

### Bioinformatics Analysis

Raw data from the MS analysis was searched against NCBI *S. aureus* MSSA476^2^ using the Sequest algorithm in Proteome Discoverer version 2.1 (Eng et al., 1994). Data were filtered to a 1% peptide and protein level false positive rate using a reverse database approach (Elias and Gygi, 2007). The area under the curve (AUC)-based label free quantitation of detected proteins was determined through the Proteome Discoverer workflow. Proteins commonly found in the two groups (LB and BHI) and unique to each group were classified according to biological processes, molecular functions, and cellular components using DAVID (Huang et al., 2009), respectively. Benjamini–Hochberg corrected *p*-value < 0.05 and an FDR < 5% were accepted for gene ontology (GO) terms. The computational prediction of the subcellular localization of MV proteins was performed by PSORTb^3^ (Yu et al., 2010). For the appropriate input, GI numbers were converted to the UniProt ID. In total, 373 out of 639 MVs proteins were mapped into UniProtKB ID. The breakdown by localization is graphed as a pie chart. The UniProt ID was utilized to extract the protein sequence. The lipoprotein, and secretory signal peptides were predicted using PRED-LIPO^4^ (Bagos et al., 2008).

### Growth Curves of *S. aureus* MSSA476 in the Presence and Absence of Exogenous MVs

*Staphylococcus aureus* MSSA476 was grown overnight in tryptic soy broth. The next day, the culture was diluted and further grown in BHI and LB broth, washed in PBS, resuspended in BHI and LB broth and added to honeycomb plates (Bioscreen, United States) in a total volume of 200 μl. Purified MVs from *S. aureus* MSSA476 grown in LB or BHI were administered at a total concentration of 20 μg (0.1 μg/μl). Growth was monitored by measuring OD_600 nm_ every 15 min under shaking conditions using Bioscreen C MBR machine (Growth Curves, United States).

### Blood Survival Assay

The viability of *S. aureus* in whole human blood was assessed and performed as previously described (Askarian et al., 2017). Briefly, blood from healthy donors was collected in tubes containing hirudin (lepirudin; Roche, Switzerland), which is a specific inhibitor of thrombin thereby preventing coagulation without impairing complement activity. Approximately 160 μl of freshly drawn blood was mixed with 20 μl of *S. aureus* MSSA476 (∼8 × 10^6^ CFU/ml) or MRSA USA300 (∼1 × 10^6^ CFU/ml) in RPMI 1640 (Gibco, Life Technologies, United Kingdom) containing 0.05% human serum albumin (HSA; Sanquin, Netherlands). Thereafter, 20 μl of buffer (RPMI/HSA) or 5–20 μg MVs (0.025–0.1 μg/μl) in buffer was added to the blood with bacteria-samples. When indicated, the MVs were sonicated, left untreated or treated with 0.1 μg/ml proteinase K (PK; Life Technologies, United Kingdom) for 3 h at 56°C prior to use. When indicated, the blood assay was performed in the absence of MVs, presence of intact, sonicated or sonicated-PK treated MVs for 3 h at 37°C on a rotator. Blood cells were lysed by adding 800 μl ice-cold H_2_O supplemented with 0.3% saponin (Sigma–Aldrich, Germany). Bacterial survival in blood was evaluated by serial dilution of blood with subsequent plating on blood/Todd Hewitt agar (THA) plates following overnight incubation at 37°C. The percentage of bacterial survival was determined by comparing surviving bacteria to the input inoculum.

### Neutrophil Isolation

Neutrophils were isolated from the heparinized venous blood of healthy volunteers (see section “Ethical Approval”) using 1-Step polymorphprep (Fresenius Kabi Norge AS, Norway) gradient centrifugation.

### Neutrophil Killing Assay

The assay was performed as previously described using freshly isolated neutrophils (Askarian et al., 2017). Briefly, bacteria were opsonized with 5% serum and incubated with neutrophils in RPMI/HSA at an MOI = 1.5 or MOI = 10. Thereafter, 20 μg of MVs (0.1 μg/μl), purified from bacteria grown in LB or BHI was added to the assay when indicated. The neutrophils and bacteria, in presence or absence of MVs were incubated at 37°C on a rotator. After 45 min, neutrophils were lysed using 800 μl of ice cold H_2_O supplemented with 0.3% saponin followed by 5 min incubation on ice. Surviving bacteria were quantified following serial dilution and plating on THA plates. The percentage of bacterial survival was determined by comparing the numbers of surviving bacteria to the input inoculum.

### Whole Blood Phagocytosis

Fluorescent-labeling of bacteria was performed using 0.5 mg/ml FITC (Sigma–Aldrich, Germany) as previously described (Askarian et al., 2017). Next, 20 μl of FITC-labeled *S. aureus* MSSA476 (∼1 × 10^8^ CFU/ml) was incubated for 15 min at 37°C with 160 μl of freshly isolated human blood anticoagulated with hirudin in RPMI/HSA. When indicated, 20 μg of purified MVs (0.1 μg/μl) were added to the samples. The red blood cells were lysed by adding FACS lysing solution (BD Biosciences, United States). The remaining cells (primarily neutrophils) were washed and analyzed by flow cytometry (BD Biosciences, United States). The fluorescence intensity of 10,000 gated neutrophils was measured for each sample. The geometric mean of the fluorescence intensity was calculated using FlowJo software.

### Murine Model of Intravenous Infection

An established murine model of *S. aureus* systemic infection (Askarian et al., 2014) was utilized to identify the role of MVs in promoting *S. aureus* survival in blood. Eight-week-old male C57BL/6 mice (*n* = 10/group; Charles River, Wilmington, MA, United States) were infected intravenously with approximately 2 × 10^8^ CFU *S. aureus* MSSA476 by tail vein injection. PBS or MVs isolated from *S. aureus* MSSA476 grown in BHI (50 μg/mice) was added to the bacteria resuspended in PBS and incubated for 30 min prior to the injection of mice. The bacterial load in blood (CFU/ml) was quantified at 24 h postinfection by plating serial dilutions on THA.

### Examination of Cell Viability

HaCaT cells, THP-1 cells, and neutrophils were seeded in either 12- or 96-well plates (Corning, United States) at confluent concentrations. Cells were left untreated or treated with isolated MVs. Thus, 5–20 μg MVs (0.025–0.1 μg/μl) were added to neutrophils and THP-1 cells or 100 μg MVs (0.1 μg/μl) was added to the HaCaT cells. The final volume was 200 and 1,000 μl in 96- and 12-well plates, respectively. Cell culture supernatants were collected at the time points indicated and centrifuged to pellet the cellular debris. Cellular cytotoxicity was assessed by measuring the levels of lactate dehydrogenase (LDH; Promega, United States) released by host cell into supernatant. As an additional approach to visualize cell death, THP-1 cells and neutrophils were stained with PI, which cannot penetrate the intact host cell membrane. Representative live imaging was performed by the Zeiss AxioObserver D1 microscope (Zeiss, Germany).

### NET Induction Assay

Freshly isolated neutrophils were seeded in 96-well plates at a density of 2 × 10^5^ cells/well in Hank’s balanced salt solution (HBSS) supplemented with calcium and magnesium. The cells were left untreated or treated with 20 μg MVs (0.1 μg/μl) and incubated for at least 3 h at 37°C with 5% CO2. Additionally, the chemical neutrophil extracellular trap (NET) inducer phorbol 12-myristate 13-acetate (PMA; Sigma–Aldrich, Germany) was added to wells where applicable. Micrococcal nuclease was then added to the wells before an additional 10 min incubation at 37°C. Micrococcal nuclease activity was stopped by the addition of 5 mM EDTA to each well, and plates were centrifuged at 200 × *g* for 8 min prior to collecting supernatant. The extracellular DNA content in the supernatant was then quantified using a Quant-IT PicoGreen dsDNA Assay kit (Life Technologies, CA, United States) according to the manufacturer’s instructions.

### NET Visualization by Fluorescence Microscopy

Neutrophils were seeded in 96-well plates at a density of 5 × 10^4^ cells/well. NET production was induced as described above and the cells were fixed by the addition of paraformaldehyde (final concentration of 4%) followed by a 20 min incubation at room temperature. The cells were blocked with PBS supplemented with 2% bovine serum albumin and 2% goat serum. Thereafter, the cells were immune-stained using rabbit anti-human myeloperoxidase primary antibody, Alexa Fluor 488 goat anti-rabbit immunoglobulin G secondary antibody (Life Technologies, United States) and Hoechst-33342-trihydrochloride (Life Technologies, United States) as previously described (Corriden et al., 2015). Imaging was performed by the Zeiss AxioObserver D1 microscope (Zeiss, Germany).

### Degranulation of Neutrophils Quantified by Elastase Release

The elastase release was performed as previously described with minor modifications (Fuchs et al., 2007). Briefly, 2 × 10^5^ neutrophils/well were resuspended in HBSS supplemented with calcium and magnesium, and seeded into each well of a 96-well plate. The cells were left untreated or treated with 20 μg MVs (0.1 μg/μl) or 0.02% Triton X-100, which served as a positive control (Fuchs et al., 2007). The plate was incubated for 1 h at 37°C with 5% CO2 and centrifuged at 200 × *g* for 8 min. Thereafter, degranulation was measured by incubating 100 μl of supernatant with the elastase substrate, *N*-(methoxysuccinyl)-Ala-Ala-Pro-Val 4-nitroanilide (Sigma–Aldrich, Germany), for 30 min followed by measurement of optical density 405 nm using a Multimode Plate Reader (PerkinElmer, Enspire Alpha, United States).

### ROS Production Assay

The reactive oxygen species (ROS) production assay was performed as previously described (Corriden et al., 2015). Briefly, 2 × 10^6^ neutrophils/ml were incubated in buffer (HBSS without calcium and magnesium; Gibco, Life Technologies, United Kingdom) containing 10 mM 2′,7′-dichlorofluorescin diacetate (Sigma–Aldrich, Germany) for 20 min at 37°C with gentle agitation. The neutrophils now containing a cell permeable non-fluorescent probe were then centrifuged at 400 × *g* for 5 min and washed with buffer. The neutrophil suspension was added to a flat bottom 96-well plate (2 × 10^5^ cells/well). Cells were then left untreated or treated with 20 μg MVs (0.1 μg/μl) or PMA (positive control). Oxidation of the probe results in fluorescence (product information Sigma–Aldrich), and the fluorescence intensity (485 nm excitation, 530 nm emission) was measured at 15 min intervals using a Multimode Plate Reader over the course of 2 h.

### *In Vitro* Production of Cytokines from Eukaryotic Cells

The effect of purified MVs on the cytokine secretion by HaCaT and THP-1 cells was evaluated using Bio-Plex Pro^TM^ Human Cytokine 27-plex Assay (Bio-Rad, United States; Henno et al., 2017) and Quantikine ELISA kit (R&D Systems Inc., United States), respectively. Briefly, 1.5 × 10^6^ HaCaT cells were seeded in 12-well plates (Corning, United States) to the final volume of 1,000 μl and 1 × 10^5^ THP-1 cells were seeded in 96-well plates (Corning, United States) to the final volume of 200 μl. HaCaT (100 μg MVs, i.e., 0.1 μg/μl), THP-1 (20 μg MVs, i.e., 0.1 μg/μl) and freshly purified neutrophils (5–20 μg MVs, i.e., 0.025–0.1 μg/μl) were treated with MVs isolated from *S. aureus* MSSA476 grown in LB or BHI media or left untreated. Thereafter, the cells were incubated at 37°C in a CO_2_ incubator with 5% CO_2_.

The culture supernatants were collected at the time points indicated and centrifuged at 4°C 13,000 × *g* for 7 min to pellet the cellular debris. The cytokines secreted into the culture supernatants were measured using multiplex and ELISA kits according to the manufacturer’s instructions. The range of cytokine response and detection measured are presented in Supplementary Figure 4.

### Immunization of Mice with *S. aureus*-Derived MVs

A murine model was employed to determine if *S. aureus*-derived MVs are immunogenic facsimiles of their parental bacteria. Ultimately, *S. aureus* MRSA (USA300) was chosen as a model organism as we previously encountered difficulties with inducing sustained bacteremia and death using MSSA476 in BALB/c mice. Additionally, *S. aureus* MRSA (USA300) has been well characterized to have strong immune evasive properties and poses various clinical challenges (Pier, 2013). First, MVs production by the MRSA was confirmed via TEM analysis (results not shown). Next, 8-week-old female BALB/c mice (Charles River, Wilmington, MA, United States) were injected intraperitoneally (i.p.) with purified membrane vesicles obtained from *S. aureus* USA300 MRSA grown in BHI broth at the concentration of 100 μg/mice at week 1, followed by 50 μg/mice at weeks 2 and 3 (*n* = 10; **Figure 4A**). The control mice were treated with PBS (*n* = 10). One week after the last immunization, mice were bled submandibularly. Lastly, sera were collected, pooled and stored at -80°C for analysis of antibody titers by ELISA.

### Antibody Response of Immunized Mice

The serum antibody response was measured 7 days following the last immunization in control (i.e., PBS treated) and MV immunized mice. Briefly, microtiter plates (Nunc, Denmark) were coated with 30 μg MVs in coupling buffer (100 mM carbonate/bicarbonate buffer pH 9.6, Sigma–Aldrich, Germany) and incubated overnight at 4°C. Wells were blocked with blocking buffer consisting of 1% (w/v) HSA for 2 h at 37°C and washed with PBS supplemented with 0.05% (v/v) Tween 20. Thereafter, 100 μl of diluted serum in pre-warmed blocking buffer were loaded in the plate and incubated for 1 h at room temperature. Secondary HRP-conjugated anti-mouse IgG2a, IgG2b, IgG1, and IgA (Southern Biotechnology Associates, United States), as well as IgM and IgG (Bethyl, United States) were added to the plate wells (100 μl of a 1/5,000 dilution in blocking buffer) and the plates were incubated for 1 h at 37°C. The Multimode Plate Reader was then used to measure absorbance at 450 nm.

### Murine Model of Subcutaneous and Intraperitoneal Infection

We employed established *in vivo* murine models of systemic and skin infection to determine if immunization with *S. aureus* derived-MVs was protective against a live bacterial challenge. For systemic infection, the PBS (control)- or MV-immunized mice were infected i.p. with 2 × 10^9^ CFU bacteria in exponential phase (OD_600_ = 0.6). Observed mortality was recorded twice per day up to 10 days postinfection. For the subcutaneous infection model, PBS- or MV-immunized mice were infected intradermally on a shaved flank with 2 × 10^8^ CFU bacteria in the exponential phase (OD_600_ = 0.6). Area measurements (mm^2^) of lesions (defined as dermonecrotic areas) were carried out on day 3 postinfection. Abscesses were excised, homogenized, serially diluted in PBS and bacterial loads (CFU/abscess) were quantified.

The degree of protective immunity in each respective model was determined by survival of the mice or enumerating the bacterial load in immunized and unimmunized mice.

### Ethical Approval

Human neutrophils were isolated from freshly drawn human blood. All blood donors provided written informed consent. Additionally, all human blood draws and *ex vivo* analyses were performed in accordance with the ethical principles outlined in the Helsinki Declaration, the UCSD Medical Ethics Committee, and ethical approval of 2014/1653 REK North-Norway. Animal studies were performed under the UCSD approved IRB protocol S00227M, and in accordance with the rules and regulations outlined by the UCSD Institutional Animal Care and Use Committee.

### Statistical Analysis

Data from *in vitro*/*ex vivo* assays were represented as the means ± standard error of mean (SEM) of at least three independent experiments. The data presented in **Figure 2D** is expressed as mean ± SEM of two independent experiments each performed in triplicate. Data from the *in vivo* experiments expressed as mean ± SEM represent one experiment performed with 10 mice/group (mean ± SEM). The statistical analysis was performed using pooled data for each experiment. Student’s *t*-test or two-way ANOVA was used for determination of statistically significant differences between groups (*P* < 0.05). Graphs were generated using Excel or Graph Pad Prism.

## Results

### *S. aureus*-Derived MV Varies in Size and Associated Proteins Depending on Growth Conditions

**FIGURE 1:**
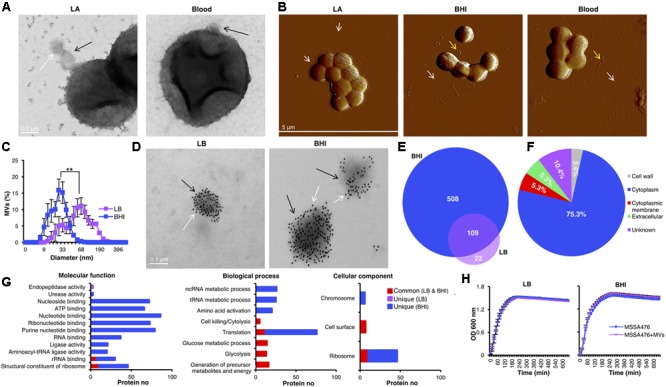
The size and protein cargo of *S. aureus*-derived MVs were markedly different depending on growth conditions. **(A)** TEM-immunogold analysis of MV-generation on *S. aureus* MSSA476 (black arrow) grown on LA and blood agar plate. Gold particles (white arrows) indicate the presence of peptidoglycan/peptidoglycan-precursors. The scale bar is shown. **(B)** Atomic force micrographs of *S. aureus* MSSA476 cultivated on LA, BHI- and blood agar plates. Arrows indicate MVs on the bacterial surface and the released MVs. The scale bar is shown. **(C)** The average size distribution of MVs was determined using dynamic light scattering. The data are expressed as mean ± SEM (standard error of the mean) of four independent measurements performed on independent samples. **(D)** TEM-immunogold labeling of peptidoglycan/peptidoglycan-precursors in purified vesicles (black arrows) obtained from *S. aureus* MSSA476 grown in BHI and LB purified by flotation through an OptiPrep density gradient. Gold particles (white arrows) indicate the presence of peptidoglycan/peptidoglycan-precursors. Scale bars are shown. **(E)** *S. aureus*-derived vesicles from MSSA476 grown in LB and BHI were purified by flotation through an OptiPrep density gradient. Venn diagram illustrates common and unique proteins associated with MV identified using LPI/in-solution approaches. **(F)** Localization of the MV-associated proteins shown as the percentage in a pie chart. **(G)** Histograms representing the distribution of identified vesicular proteins according to their molecular functions, role in biological processes and cellular components. **(H)** Supplementation of MVs (20 μg of total MV, i.e., 0.1 μg/μl) in the culture media did not influence bacterial growth in LB media (left panel) or BHI (right panel). Data represent as the mean ± SEM of three independent experiments. The significance is indicated by asterisks (^∗^): ^∗∗^*P* ≤ 0.01.

A TEM-immunogold staining of the methicillin-sensitive hypervirulent community-acquired *S. aureus* strain MSSA476 demonstrated MV-release from bacteria grown *in vitro* on LA and blood agar plates (**Figure 1A**). The gold particles (shown as black dots) indicate the presence of peptidoglycan/peptidoglycan precursors (white arrows) on the released MVs (black arrows) from the bacterium. Different growth conditions influence the expression of *S. aureus* virulence factors (Oogai et al., 2011; Chaves-Moreno et al., 2016). However, whether changing growth environment could influence the *S. aureus* MV cargo and functions remain elusive, and these were our research questions. Thus, MVs were studied in bacteria grown on enriched medium containing host components such as hemoglobin (BHI and blood agar) and a simpler medium (LB). First, AFM was performed to analyze MV release from *S. aureus* strain MSSA476 grown on LA, BHI and blood agar plates. MVs from *S. aureus* grown on LA plates remained associated with the bacterial surface but were also found in the surrounding environment (**Figure 1B**, left panel and Supplementary Figure 5). Interestingly, the growth of *S. aureus* on BHI or blood agar yielded an abundance of MVs primarily in the surrounding environment (white arrow) or as accumulated aggregates (yellow arrow; **Figure 1B**, middle and right panels).

The size distributions of *S. aureus* MVs derived from bacteria grown in both media were measured by dynamic light scattering. The average size of MVs isolated from LB-grown bacteria (46.6 ± 5.9 nm) was twofolds larger than MVs isolated from the BHI-grown bacteria (24.4 ± 2.8 nm) (*P* < 0.05; **Figure 1C**). Notably, MVs from the LB-grown bacteria also had a small population of MVs with similar size as observed for MVs produced by bacteria grown in BHI. Collectively, these results demonstrate that the growth media can influence the size distribution of MVs produced by *S. aureus*.

Next, we examined and compared the effect of different growth conditions on MV-associated proteins. MVs isolated from *S. aureus* grown in LB or BHI was further purified using a differential centrifugation gradient to remove cellular debris and contaminating proteins. Fractions were taken from the gradient and analyzed on Coomassie Blue-stained SDS PAGE. The fractions showing the same protein profile were pooled (examples are shown in Supplementary Figure 2) and the MV purity was verified by TEM negative staining (examples are shown in Supplementary Figure 3A) and AFM imaging (examples are shown in Supplementary Figure 3B). The quality/purity of the MV preparation was also tested by immunogold labeling using an antibody against the peptidoglycan epitope (**Figure 1D**). Our results demonstrated purified MVs are densely covered with peptidoglycan or peptidoglycan-precursors (**Figure 1D**). More gold particles were detected in the purified MVs (**Figure 1D**) compared to MVs released during *in vitro* bacterial culture on LA or blood agar plates (**Figure 1A**), as the whole bacterial cells likely compete with MVs to capture more peptidoglycan antibody.

Proteomic analysis was performed using an In-sol and a LPI approach. The latter approach was utilized to increase the chance of detecting surface-exposed proteins (Chooneea et al., 2010; Karlsson et al., 2012). The resulting MS/MS spectra of peptides identified in the *S. aureus* MVs were searched against the genome of *S. aureus* MSSA476. A total of 131 and 617 proteins were identified in the MVs isolated from *S. aureus* grown in the LB and BHI broth, respectively (Supplementary Data Sheet 1). A comparative proteomic analysis was performed to identify common or unique proteins associated with both pools of *S. aureus* MVs. A total of 109 common proteins were identified in the MSSA476 MVs isolated from both cultures, whereas 22 and 508 proteins were uniquely associated with the MVs from bacteria grown in LB vs. BHI, respectively (**Figure 1E**). The overall distribution of expressed proteins in MVs (Supplementary Figure 6 and Supplementary Data Sheet 2) revealed that 46 of the identified proteins were found in MVs isolated from both growth conditions and detection approaches (Supplementary Figure 6, ABCD). However, all other proteins were exclusively associated with one particular media condition and/or identification method; 277 (A, BHI/LPI), 36 (B, BHI/in solution), 9 (C, LB/LPI), and 7 (D, LB/in solution) proteins (Supplementary Figure 6 and Supplementary Data Sheet 2).

Several known virulence-associated proteins and toxins were identified in both pools of MVs (**Table 1** and Supplementary Data Sheets 1, 3). A quantitative proteomic analysis was performed using the AUC to assess peptide abundance. Formate acetyltransferase and delta-hemolysin precursor were identified as the most abundant proteins in the *S. aureus* MVs isolated from LB and BHI, respectively (Supplementary Data Sheet 1). An *in silico* approach was employed to identify lipoproteins and secretory signal peptides in *S. aureus*-derived MVs. A large number of detected proteins lack the signal peptide and lipobox motif (Supplementary Data Sheet 3).

**Table 1:** Virulence factors associated with MVs isolated from *S. aureus* grown in BHI and LB.

GI accession no.	Protein names	Gene names	Media
49243703	Alkyl hydroperoxide reductase subunit F	*ahpF*	BHI
49243704	Alkyl hydroperoxide reductase subunit C	*ahpC*	LB and BHI
49244335	Autolysin	*atl*	LB and BHI
49244621	Catalase	*katA*	LB and BHI
49244675	Conserved virulence factor B	*cvfB*	BHI
49245914	Collagen adhesin	*cna*	LB
49244100	Clumping factor A	*clfA*	LB and BHI
49245852	Clumping factor B	*clfB*	LB
49244762	Elastin-binding protein	*ebpS*	BHI
49244091	Enolase	*eno*	LB and BHI
49245254	Enterotoxin	seq	LB and BHI
49245208	Enterotoxin type A	Sea	LB
49245255	Staphylococcal enterotoxin Sek	Sek2	LB
49245155	Ferritin	*ftnA*	LB and BHI
49245375	Putative non-heme iron-containing ferritin	–	BHI
49245643	Immunoglobulin-binding protein sbi	*sbi*	LB and BHI
49243431	Immunoglobulin G binding protein A precursor	*Spa*	LB and BHI
49244444	α-Hemolysin	*hly/hla*	LB
49245273	Delta-hemolysin	*hld*	LB and BHI
49245646	Gamma-hemolysin component A	*hlgA*	LB and BHI
49245645	Gamma-hemolysin component C	*hlgC*	LB
49245258	Putative leukocidin S subunit	*lukS*	LB and BHI
49245257	Putative leukocidin F subunit	SAS1924 (*lukF*)	LB and BHI
49245789	*O*-acetyltransferase OatA	oatA	BHI
49244410	Iron-regulated surface determinant protein	*isdA*	LB and BHI
81696343	Iron-regulated surface determinant protein	*isdB*	LB and BHI
49245791	Immunodominant staphylococcal antigen A	*isaA*	BHI
49245611, 49243657	MarR family transcriptional regulator	*marR*	BHI
49245860	Immunodominant staphylococcal antigen B	*isaB*	LB
49244456	Phenol-soluble modulin beta 1	*psmB1*	BHI
49244216	Regulatory protein Spx	*spxA*	BHI
49244438	Extracellular fibrinogen binding protein	*efb*	LB and BHI
49243599	Peptidoglycan hydrolase	*lytM*	BHI
49245204	Staphylokinase	*sak*	LB
49243933	Staphylococcal accessory regulator A	*sarA*	BHI
49245519	Staphylococcal accessory regulator R	*sarR*	BHI
49243432	Staphylococcal accessory regulator S	*sarS*	BHI
49245749	Sortase A	*srtA*	BHI
49244001	HTH-type transcriptional regulator MgrA	*mgrA*	BHI
49243871	Bone sialo binding protein	*bbP*	LB and BHI
81696368	Serine-aspartate repeat-containing protein D	*sdrD*	LB
49245171	Staphopain A	*sspP*	LB
49244330	Staphopain B	*sspB*	LB and BHI
81649004	Staphylococcal complement inhibitor (SCIN)	*sciN*	LB
49245302	Serine-protein kinase RsbW (anti-sigma B factor)	*rsbW*	BHI
49245304	Putative sigma factor sigB regulation protein	*rsbU*	BHI
49244832	Superoxide dismutase	*sodA*	BHI
49243605	Staphylococcal Esx proteins (EsxA)	*esxA*	BHI
49245523	Staphylococcal secretory antigen ssaA2	ssaA2	BHI
49243983	HTH-type transcriptional regulator SarX	*sarX*	BHI
49243983	HTH-type transcriptional regulator rot	*rot*	BHI

Identified MV proteins were classified according to their estimated location in the bacterial cell (**Figure 1F** and Supplementary Data Sheet 3). Cytoplasmic proteins constituted the most abundant detected protein class (75.33%), followed by proteins of unknown localization (10.4%)-, cytoplasmic membrane proteins (5.3%)-, extracellular or secreted proteins (5.3%), and cell wall-associated proteins (3.4%; **Figure 1F**). Molecular functions, biological processes and cellular components associated with the identified MV proteins were categorized according to GO functions using the DAVID server (Huang et al., 2009). According to molecular functions, the majority of unique MV proteins isolated from bacteria grown in BHI possessed either potential DNA/RNA/ATP binding activities or ligase activities, while proteins from LB-derived MVs were enriched for endopeptidase activity (**Figure 1G**, left panel). Various proteins found in both pools of MVs were involved in crucial metabolic or virulence processes such as carbohydrate synthesis or host cell cytolysis, while a major component of unique MV proteins isolated from BHI was associated with protein translation (**Figure 1G**, middle panel). Moreover, the proteins within *S. aureus*-derived MVs could be associated with different cellular components such as the chromosome, cell surface, or ribosome (**Figure 1G**, right panel). A significant portion of unique MV proteins isolated from BHI was associated with ribosomes (**Figure 1G**, right panel). Despite the presence of several proteins involved in bacterial metabolism within MVs, media supplementation with an exogenous source of purified MVs did not influence MSSA476 growth (**Figure 1H**).

In summary, all these results demonstrated different growth conditions influence the size distribution and proteins associated with MVs produced by *S. aureus*.

### Exogenous MVs Increased Resistance of *S. aureus* to Whole Blood and Neutrophil Killing

**FIGURE 2:**
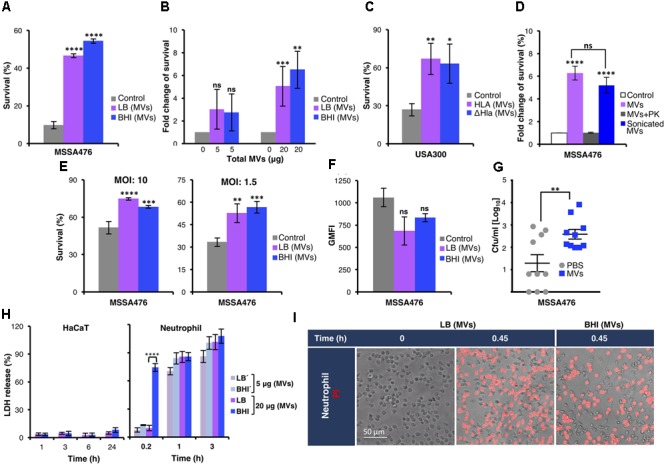
*Staphylococcus aureus* MVs promote bacterial survival in human whole blood and in the presence of neutrophils *ex vivo* and *in vivo*. **(A)** Survival of *S. aureus* MSSA476 in blood is increased in the presence of 20 μg MV (0.1 μg/μl) isolated from MSSA476 grown in LB and BHI [marked as LB (MVs) or BHI (MVs) in the figure] compared to absence of MVs (marked as control in the figure). The number of inoculated bacteria at time point 0 was set to 100% and the number of surviving bacteria after 3 h is represented as the percentage of inoculation. **(B)** *S. aureus* MSSA476 survival in blood is increased in the presence of MVs, in a dose-dependent manner (5–20 μg of total MVs, i.e., 0.025–0.1 μg/μl). The number of surviving bacteria after 3 h in the absence of MVs was arbitrary set as 1, and the number of surviving bacteria in the presence of MVs is represented as the fold change compared to bacteria in the absence of MV. **(C)** Survival of USA300 MRSA in human blood is increased in the presence of MVs isolated from USA300 (MV-Hla) and USA300ΔHla (MV-ΔHla) grown in BHI. The percentage of survival was calculated as described in **(A)**. **(D)** Sonication of purified MVs followed by proteinase K (PK) treatment abolished the effect of MVs on bacterial survival in human whole blood. The fold change of survival was calculated as described in **(B)**. **(E)** Survival of opsonized *S. aureus* MSSA476 in the presence of neutrophils is enhanced by supplementation of MVs isolated from bacteria growing in LB and BHI. Percentage of survival was calculated as described in **(A)**. **(F)** *S. aureus* MSSA476 were labeled with FITC and incubated with human whole blood in the absence or presence of MVs isolated from MSSA476 grown in LB and BHI. Data represents geometric mean of the fluorescence intensity (GMFI). **(G)** Bacterial loads in the blood (CFU/ml) of 8-week-old C57BL/6 mice were counted 24 h after the mice were intravenously infected with *S. aureus* MSSA476 supplemented with PBS or an exogenous source of MVs isolated from MSSA476 grown in BHI. **(H)** HaCaT (100 μg of total MVs, i.e., 0.1 μg/μl) and freshly purified neutrophils were treated with MVs (5–20 μg of total MVs, i.e., 0.025–0.1 μg/μl) isolated from *S. aureus* MSSA476 grown in LB or BHI at the time points indicated. Percentage of cytotoxicity was calculated by measuring the amount of LDH released from the cytosol of damaged cells into the supernatant after exposure to MVs. **(I)** Viability staining of neutrophils in the presence (20 μg of total MVs, i.e., 0.1 μg/μl) or absence of MVs were performed using propidium iodide (PI). Live imaging was performed after 0 and 0.45 or 1.5 h using fluorescence microscopy. Scale bar is shown. The data represent as the mean ± SEM of at least three independent experiments except for **(D)**, which the data are expressed as the mean ± SEM of two independent experiments performed in triplicate. Mice study corresponds to one experiment performed with 10 mice/group. The significance is indicated by asterisks (^∗^): ^∗^*P* < 0.05; ^∗∗^*P* ≤ 0.01; ^∗∗∗^*P* ≤ 0.001; ^∗∗∗∗^*P* ≤ 0.0001. ns, no significant difference.

Both pools of MVs contained several virulence factors (**Table 1**) with proven or postulated roles in promoting *S. aureus* survival in human blood (Malachowa and DeLeo, 2011; Thomer et al., 2016). To test this experimentally, *S. aureus* was incubated in 80% freshly drawn human blood in the absence or presence of exogenously administered MVs. As shown in **Figure 2A**, MVs strongly promoted *S. aureus* MSSA476 survival in blood, in a dose-dependent manner (**Figure 2B**). The MV-mediated increase in bacterial survival in human blood was confirmed in the methicillin-resistant *S. aureus* USA300 (**Figure 2C** and Supplementary Figure 7A). Moreover, the increased survival in blood was independent of α-hemolysin (Hla) (**Figure 2C** and Supplementary Figure 7A), one of the pore-forming toxins identified in the proteomic analysis (**Table 1** and Supplementary Data Sheet 1). Next, we evaluated whether MV-associated proteins are required for the observed MV-mediated increased bacterial survival. In additional experiments, MVs were sonicated and left untreated, or treated with PK to degrade MV-associated proteins before being used as a supplement in whole blood assay. As shown in **Figure 2D** and Supplementary Figure 7B, the MVs with intact proteins increases bacterial survival, while PK treated MVs completely abolished the protective effect previously observed. These results underscore the ability of the MV cargo to protect the pathogen from being killed by components of human blood.

We next assessed whether *S. aureus* MVs confer resistance to neutrophils, the most abundant leukocyte present in whole blood. *S. aureus* was then co-incubated with purified human neutrophils in the absence or presence of exogenously administered MVs derived from bacteria grown in LB or BHI. Once again despite differences in the MV protein content, both pools of MVs significantly promoted resistance of *S. aureus* to human neutrophil killing (**Figure 2E**). MV promotion of *S. aureus* survival in blood could not be explained by differences in neutrophil phagocytosis *per se*, as uptake of *S. aureus* by human neutrophils within whole blood was comparable in the presence and absence of MVs when assessed by flow cytometry (**Figure 2F**). Currently, no *S. aureus* strain deficient in the ability to generate MVs is known. Therefore, we employed a murine intravenous infection model in the presence or absence of supplemental MVs to evaluate the influence of MV on bacterial survival *in vivo* by assessing the bacterial load from blood and homogenized kidney, spleen, and liver 24 h postinfection. As shown in **Figure 2G**, increased *S. aureus* recovery was observed from mice infected with bacteria supplemented with MVs compared to the control. In contrast, bacterial loads in the kidney, spleen, and liver did not differ significantly between mice infected with *S. aureus* supplemented with MV compared with mice infected with *S. aureus* alone (Supplementary Figure 8).

These findings suggest exogenous MVs increase bacterial survival in whole blood and within neutrophils. Our proteomic analysis indicated that *S. aureus* MVs contained several cytolysins and toxins (**Table 1** and Supplementary Data Sheet 1) with the potential for cytotoxic activity against host immune cells. We next explored whether *S. aureus* MVs could influence neutrophil viability and subsequently increase resistance to neutrophil killing. Cytotoxicity of *S. aureus* MVs to neutrophils was evaluated *in vitro* through LDH release at various time points (**Figure 2H**). The viability of human neutrophils decreased dramatically following even short-term exposure to low concentrations of *S. aureus*-derived MVs (0.025 or 0.1 μg total protein/μl; **Figure 2H**, right panel). MVs with high protein diversity, derived from *S. aureus* grown in BHI, resulted in significantly higher cytotoxicity in neutrophils following brief exposure (0.2 h) to MVs (0.1 μg total protein/μl) compared to those derived from LB culture. However, longer exposures to both sources of MVs were associated with comparable levels of cytotoxicity as measured by LDH released (**Figure 2H**, right panel). The marked reduction in viability of neutrophils exposed to *S. aureus*-derived MVs were also confirmed by fluorescence microscopy using PI cell viability staining (**Figure 2I**). Remarkably, the MVs also promote extensive MV-induced cell death in the macrophage THP-1 cell lines (Supplementary Figure 9), while it exhibited minimal cytotoxicity to keratinocytes (HaCaT) even after 24 h exposure (**Figure 2H**, left panel).

Together these results suggest exogenously added MVs containing intact proteins improves the survival of *S. aureus* in the presence of blood and purified neutrophils.

### *S. aureus*-Derived MVs Possess Neutrophil-Activating Properties

**FIGURE 3:**
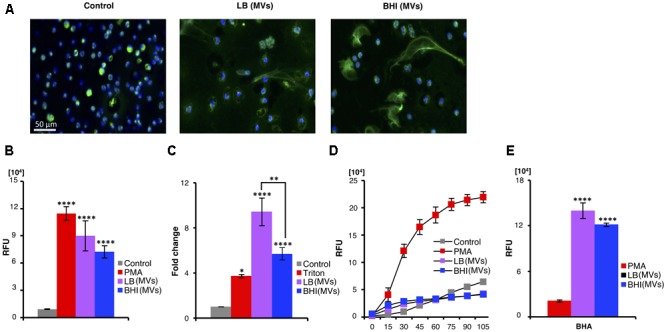
*Staphylococcus aureus* MVs promote extracellular trap formation in human neutrophils *in vitro* independent of ROS generation. Neutrophils were incubated with PBS (control) or MVs isolated from bacteria grown in LB or BHI. NET induction was evaluated using various approaches: **(A)** Immunostaining using a primary antibody against myeloperoxidase (green). The nucleus is stained with Hoechst (blue). Scale bar is shown. **(B)** Quantification of extracellular DNA and **(C)** measuring neutrophils degranulation through determining the elastase release. The absorbance at 405 nm in the absence of MVs (control) was normalized to 1, and the absorbance in the presence of MVs (LB and BHI) and the positive control (Triton) is represented as the fold change of elastase release. **(D)** DCF-based ROS assays were performed to evaluate the effect of *S. aureus* MSSA476 MVs on ROS production by neutrophils. **(E)** Neutrophils were pre-treated with the ROS scavenger BHA for 30 min before addition of either PMA or MSSA476 MVs to determine whether MVs induced NET production. Data represent as the mean ± SEM of at least three independent experiments. The significance is indicated by asterisks (^∗^): ^∗∗^*P* ≤ 0.01; ^∗∗∗^*P* ≤ 0.001; ^∗∗∗∗^*P* ≤ 0.0001.

As the *S. aureus* derived-MVs markedly affected neutrophil viability, the propensity of the MVs to induce NETs, a consequence of a specialized form of neutrophil death (Brinkmann et al., 2004), was assessed. NETs were analyzed by immunostaining of untreated (control) or MV-treated neutrophils using a primary antibody against myeloperoxidase (a NETs marker) (Papayannopoulos et al., 2010; **Figure 3A**) and by quantification of extracellular DNA release (**Figure 3B**). Both approaches demonstrated the presence of MVs induced NET release. PMA-treated and untreated neutrophils served as positive and negative controls for NET formation, respectively (**Figure 3B**). In addition, a release of the neutrophil degranulation marker elastase into the cell culture supernatant was increased upon exposure of neutrophils to *S. aureus*-derived MV compared to untreated neutrophils (**Figure 3C**). These studies revealed that MVs strongly stimulated NET production from freshly isolated human neutrophils. Next, we evaluated whether the observed MV-induced NET formation was dependent on ROS, which can be measured by oxidation of a DCFH-DA probe. The positive control, PMA, induced NET in a ROS-dependent manner, while MV-induced NET-induction appeared to be independent of ROS (**Figure 3D**). An additional approach was also employed, where the ROS formation was inhibited by the scavenger BHA. Still, MVs could induce NET formation, confirming its independence of ROS (**Figure 3E**), while BHA inhibited PMA-induced NET formation as expected (**Figure 3E**).

Our data demonstrate that *S. aureus*-derived MVs activate neutrophils independent of ROS and induce the formation of NETs.

### Mice Immunized with MVs Are Protected against *S. aureus* Challenge

**FIGURE 4:**
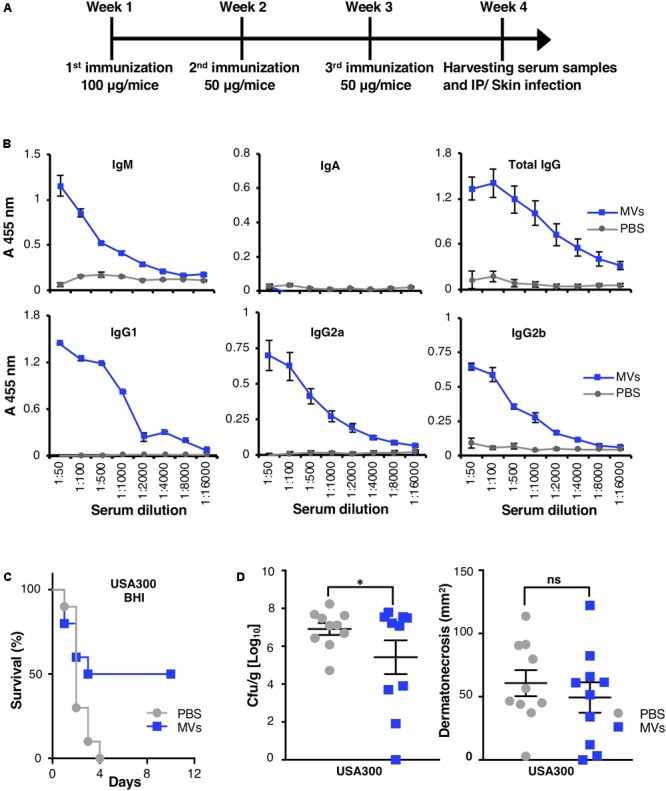
MVs-immunized mice developed IgM and IgG subclasses and were protected against live *S. aureus* MRSA challenge. MVs were isolated from MRSA grown in BHI. **(A)** The diagram represents timeline for vaccination procedure and *S. aureus* challenges. **(B)** Titers of anti-MVs IgM, IgA, IgG, and of IgG subclasses IgG1, IgG2a, and IgG2b were measured in female BALB/c mice immunized intraperitoneally with MRSA-derived MVs or with PBS. Serum was collected as depicted in **(A)**. **(C)** Percentage of survival of PBS- and MVs-immunized mice, which were challenged intraperitoneally with *S. aureus*. **(D)** Bacterial load per skin abscess (left panel) and abscess size (right panel) was measured in PBS- and MV-immunized mice 3 days after subcutaneous infection. Data correspond to one experiment performed with 10 mice/group (mean ± SEM). The significance is indicated by asterisks (^∗^): ^∗^*P* < 0.05. ns, no significant difference.

MV-associated peptidoglycan (**Figure 1D**) and multiple cell-wall attached/surface antigens (**Table 1** and Supplementary Data Sheet 1; Lee et al., 2009; Gurung et al., 2011; Jeon et al., 2016) may induce a protective antibody response. Additionally, *S. aureus*-derived MVs elicit a strong proinflammatory host response (Supplementary Figures 10, 11; Hong et al., 2011; Kim et al., 2012; Jun et al., 2017), which might provide an adjuvant effect. To study if vaccination with MVs elicited a significant host humoral immune response, mice were vaccinated i.p. with MRSA USA300-derived MVs (**Figure 4A**). No mortality was observed as a consequence of the vaccination protocol. One week after completion of the last immunization, serum was collected, and total anti-MV immunoglobulin levels were evaluated using a competitive ELISA. A sharp increase in IgM, total IgG and IgG subclasses (IgG1, IgG2a, and IgG2b) recognizing MVs was measured in the serum of MV-immunized mice compared to PBS-treated control mice (*P* < 0.05; **Figure 4B**). These finding indicate a robust antibody response to MV vaccination. Levels of IgA, an antibody involved in the immune defense of mucous membranes, remain unchanged despite intraperitoneal vaccination process (**Figure 4B**).

In order to test the effect of immunization, MV-immunized mice were challenged with *S. aureus* using established murine systemic and skin infection models. Vaccination with MVs significantly improved mouse survival and recovery after lethal challenge with USA300 MRSA (*P* < 0.05; **Figure 4C**). In a localized skin abscess infection model, bacterial loads from excised abscesses were significantly reduced in MV-immunized mice compared to control mice, 3 days postinfection (**Figure 4D**, left panel). However, no significant difference in lesion or abscess size was identified between two groups (**Figure 4D**, right panel).

In summary, immunization of mice with *S. aureus*-derived MVs elicited high anti-MV antibody titers and conferred protection against *S. aureus* infection.

## Discussion

*Staphylococcus aureus* produces spherical, bi-layered MVs during growth *in vitro* (Lee et al., 2009; Gurung et al., 2011) and *in vivo* (Gurung et al., 2011). In our study, *S. aureus* was observed to produce spherical MVs (**Figures 1A,B**) of varying size under different *in vitro* growth conditions (LB vs. BHI; **Figure 1C**), which are smaller than the Gram-negative OMVs (Lee et al., 2009; Gurung et al., 2011). Moreover, the heterogeneity within the size distribution obtained from isolated MVs from bacteria growing in LB may suggest *S. aureus* produces two different types of MVs under a nutrient-limited conditions (**Figure 1C**). The growth media also influenced proteins associated with *S. aureus*-derived MVs (**Figure 1E** and Supplementary Data Sheets 1, 2), the respective biological processes they possessed (**Figure 1G**), which was consistent with other studies (Lee et al., 2009; Gurung et al., 2011; Jeon et al., 2016). Several adhesins were identified through proteomic analysis of the *S. aureus* MVs (**Table 1**), which suggest a role for MVs in bacterial adhesion, colonization and tissue invasion. In addition, the presence of a substantial number of proteins lacking a signal peptide (Supplementary Data Sheet 3) suggests the importance of MVs for releasing *S. aureus* virulence factors and effector molecules. Although cell lysis was recently suggested as a putative mechanism for MV biogenesis in Gram-negative (Turnbull et al., 2016) and Gram-positive bacteria (Toyofuku et al., 2017), it is likely that other mechanisms of MV release also exists (Brown et al., 2015; Orench-Rivera and Kuehn, 2016). Indeed, a strong association between MV generation and bacterial viability has been demonstrated in the Gram-positive strain, *Streptococcus pneumoniae* (Olaya-Abril et al., 2014). Thus, the presence of several cytoplasmic markers, e.g., Ldh, GyrB, etc, in the MV cargo (Supplementary Data Sheets 1–3) could be due to the fact that a part of the cytoplasm is trapped during MV biogenesis. Moreover, the presence of proteins involved in peptidoglycan-based cell wall biogenesis or cell wall organization properties, e.g., FemA, FemB, LytM, etc. (**Table 1** and Supplementary Data Sheets 1–3) and detection of peptidoglycan or peptidoglycan-precursors associated with *S. aureus*-derived MVs (**Figures 1A,D**) suggests that the cell wall is altered during MVs release (Lee et al., 2009).

*Staphylococcus aureus* is a leading cause of bloodstream infection and survives in the host via multiple mechanisms avoiding clearance by components of the innate immune system (Malachowa and DeLeo, 2011). Here we found that exogenously administered MVs promoted bacterial survival in an *ex vivo* whole blood model (**Figures 2A,B**). The effect was abolished when MVs were sonicated and pre-treated with PK (**Figure 2D** and Supplementary Figure 7B), suggesting MV-associated proteins are involved in increased bacterial survival in blood. A tight association of biologically active pore-forming α-hemolysins in *S. aureus*-derived MVs and cytotoxicity has been previously demonstrated (Thay et al., 2013). α-Hemolysin, was identified using proteomic analysis of our *S. aureus*-derived MVs (**Table 1** and Supplementary Data Sheets 1, 3) and in earlier studies (Lee et al., 2009; Gurung et al., 2011). However, in our study, α-hemolysin did not influence bacterial survival in blood as USA300 treated with the MVs from the isogenic mutant survived as efficiently as the wild-type (**Figure 2C** and Supplementary Figure 5A). Many other key virulence factors are involved in the pathogenesis of *S. aureus* during bloodstream infections (reviewed in Thomer et al., 2016), and several of them were identified in the MVs (**Table 1** and Supplementary Data Sheets 1–3). These proteins may have contributed to the increased bacterial survival observed in blood. A certain level of MVs seems to be required to aid pathogen survival in blood as no significant effect was observed with low doses of MVs (5 μg; **Figure 2B**).

Purified MVs were highly cytotoxic to freshly isolated neutrophils (**Figures 2H,I**), which might be due to the abundance of known *S. aureus* leukocyte-specific toxins in the MV cargo (**Table 1**; Lee et al., 2009; Gurung et al., 2011; Jeon et al., 2016). We also found that *S. aureus*-derived MVs could induce NET formation independent of ROS generation (**Figure 3**), consistent with recent observations that this cell death pathway can sometimes be elicited without oxidative burst activation (Pilsczek et al., 2010; Corriden et al., 2015; and reviewed in Remijsen et al., 2011). The Panton–Valentine leukocidin components, LukF and LukS have previously been identified as the dominant NET inducer in the bacterial culture supernatant (Pilsczek et al., 2010). Interestingly, LukF and LukS were detected in the *S. aureus*-derived MVs through proteomic analysis (**Table 1** and Supplementary Data Sheets 1–3), which might partly explain MVs NET induction.

Both pools of MVs carry lipoproteins (Supplementary Data Sheet 3), peptidoglycan-precursors (**Figure 1D**) and cell wall-anchored proteins (**Table 1** and Supplementary Data Sheets 1–3). Purified *S. aureus* MVs derived from bacteria grown in the two different media induced comparable levels of cytokine responses. Lipoproteins yield an excessive host inflammatory response (Kovacs-Simon et al., 2011; Shahmirzadi et al., 2016), while peptidoglycan (Capparelli et al., 2011) or a combination of surface antigens (Stranger-Jones et al., 2006) has been suggested to provide protective immunity against a lethal challenge in experimental *in vivo* studies. Cytokine stimulation by *S. aureus*-derived MVs (Supplementary Figures 10, 11; Hong et al., 2011; Kim et al., 2012; Jun et al., 2017) combined with a rich cargo of various proteins/antigens (**Table 1** and Supplementary Data Sheets 1–3; Lee et al., 2009; Gurung et al., 2011; Jeon et al., 2016) and detection of peptidoglycan or peptidoglycan-precursors (**Figure 1D**) suggest that these purified cell-free components might represent a vaccine candidate. Indeed, mice immunized with MVs had increased antigen-specific IgM and IgG levels (**Figure 4B**) and showed significantly attenuated lethality and bacterial load after systemic and subcutaneous infection, respectively (**Figures 4C,D**), compared to all sham (PBS)-immunized mice, which died within 4 days in our studies.

We further evaluated IgG isotype levels, as their nature can affect the FcγRs engagement by immune cells and subsequent leukocyte activities (Nimmerjahn and Ravetch, 2005; Bournazos et al., 2015). A robust induction of IgG subclasses including IgG1, IgG2a, and IgG2b antibodies was observed in MV-immunized mice (**Figure 4B**). The role of different IgG isotypes in combating *S. aureus* infections is not well understood. However, the IgG2a isotype has been shown to provide greater protection than IgG1 or IgG2b against staphylococcal enterotoxin B-induced lethal shock in murine sepsis models (Varshney et al., 2014). IgG2a also plays an important role in complement activation and phagocytosis by neutrophils (Nimmerjahn and Ravetch, 2005; Bournazos et al., 2015). Skin application of *S. aureus*-derived MVs has been shown to promote antibody and T cell-mediated immune responses (Hong et al., 2011), and a Th1-mediated cellular response was implicated in the protection of mice against staphylococcal lung infection as result of MV immunization (Choi et al., 2015).

In summary, our study revealed that growth conditions influence the size and proteome of *S. aureus*-derived MVs. Both pools of MVs elicited similar effects on selected host cellular responses despite the clear diversity of their associated proteome. The type of host response induced by MVs is clearly influenced by bacterial strains, the amount of MVs used and the content of virulence factors (reviewed in Lee, 2012; Kaparakis-Liaskos and Ferrero, 2015; Schwechheimer and Kuehn, 2015). The comparable response induced by MSSA476 MVs isolated from bacteria grown in LB and BHI may highlight the importance of the common proteins. The MVs induced a proinflammatory response, and immunization of mice resulted in IgM and IgG responses that attenuated the burden of *S. aureus* infection.

Our findings underscore the role of *S. aureus*-derived MVs and its noxious cargo in bacterial pathogenesis, its interactions with the host immune system and their potential for further exploration as a vaccine candidate.

## Conclusion

The ubiquitous release of MVs by both Gram-positive and Gram-negative bacteria is consistent with other bacterial secretion systems. Although the mechanism of MVs biogenesis in Gram-positive bacteria is not fully understood, targeting this conserved process could be a powerful approach for altering complex host–pathogen interactions. Further investigation of bacterial MV generation under host mimicking conditions will better reveal their importance and associated virulence in human infections. MVs have immunogenic properties, which can be an advantage in vaccine design. However, several issues need to be addressed before they can be developed into therapeutic agents and vaccines.

## Author Contributions

FA, VN, and MJ designed the experiments and prepared the manuscript. FA performed the experiments. JDL performed bioinformatics analysis, while FA and AK accomplished prediction of lipoprotein and secretory signal peptides using LIPO-PED. C-MT, AK, MK, and SU assisted in mice studies. MD contributed in TEM-immunogold staining. JV assisted in imaging by fluorescence microscopy using PI staining. JC assisted in performing some of the blood assays while JAL performed cytokine analysis on HaCaT cells. SW, DG, and TM contributed to experimental design and intellectual input. All authors reviewed and approved the manuscript.

## Conflict of Interest Statement

The authors declare that the research was conducted in the absence of any commercial or financial relationships that could be construed as a potential conflict of interest.

## Footnotes

1 www.nanoxis.com

2 http://www.ncbi.nlm.nih.gov/

3 http://www.psort.org/psortb/

4 http://www.compgen.org/tools/PRED-LIPO
