# Supplementary material for: Staphylococcus aureus Membrane-Derived Vesicles Promote Bacterial Virulence and Confer Protective Immunity in Murine Infection Models
Source: Front Microbiol. 2018 Feb 20;9:262. doi: 10.3389/fmicb.2018.00262 (PMC5826277; doi:10.3389/fmicb.2018.00262)
Supplement: Supplementary file 1 [file Presentation_1.pdf]

Supplementary Material

Staphylococcus aureus Membrane-derived Vesicles Promote Bacterial Virulence and Confer Protective Immunity in Murine Infection Models

Fatemeh Askarian[1,2*], John D. Lapek Jr[3+], Mitesh Dongre[4+], Chih-Ming Tsai[2+], Monika Kumaraswamy[5], Armin Kousha[1,2], J. Andrés Valderrama[2], Judith Anita Ludviksen[6], Jorunn Pauline Cavanagh[7], Satoshi Uchiyama[2], Tom Eirik Mollnes[6,8,9,10], David J. Gonzalez[3,11], Sun Nyunt Wai[4], Victor Nizet[2,11], Mona Johannessen[1]

[+]These authors contributed equally to this work.

[*]Correspondence: Dr. Fatemeh Askarian, Research group of Host Microbe Interaction, Department of Medical Biology, Faculty of Health Sciences, UiT- The Artic University of Norway, 9037 Tromsø, Norway, e-mail: Fatemeh.askarian@uit.no.

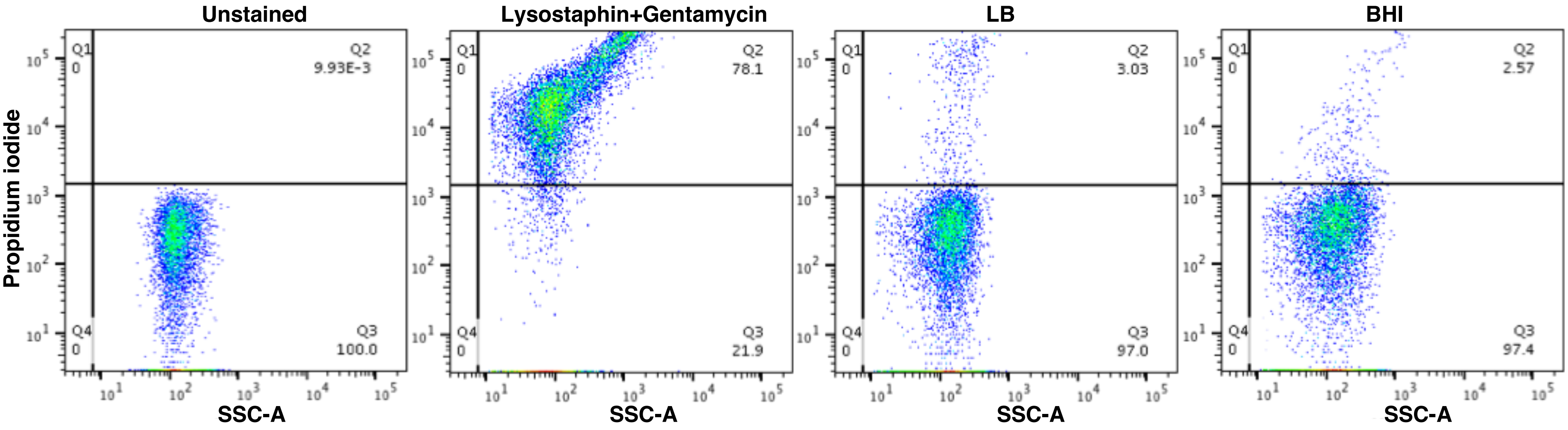

Supplementary Figure 1. Viability staining of *S. aureus* MSSA476 cells was performed using propidium iodide (PI). *S. aureus* MSSA476 was grown overnight (12h post-inoculation) at 37°C in Luria-Bertani (LB) and brain-heart infusion (BHI) broth. Bacterial cells were harvested, stained with PI and analyzed by flow cytometry. Live (PI^-, Q3) and dead (PI^+, Q2) cells are shown in the quadrant. *S. aureus* treated with gentamycin and lysostaphin, and stained with PI served as the positive control. Unstained cells served as the negative control.

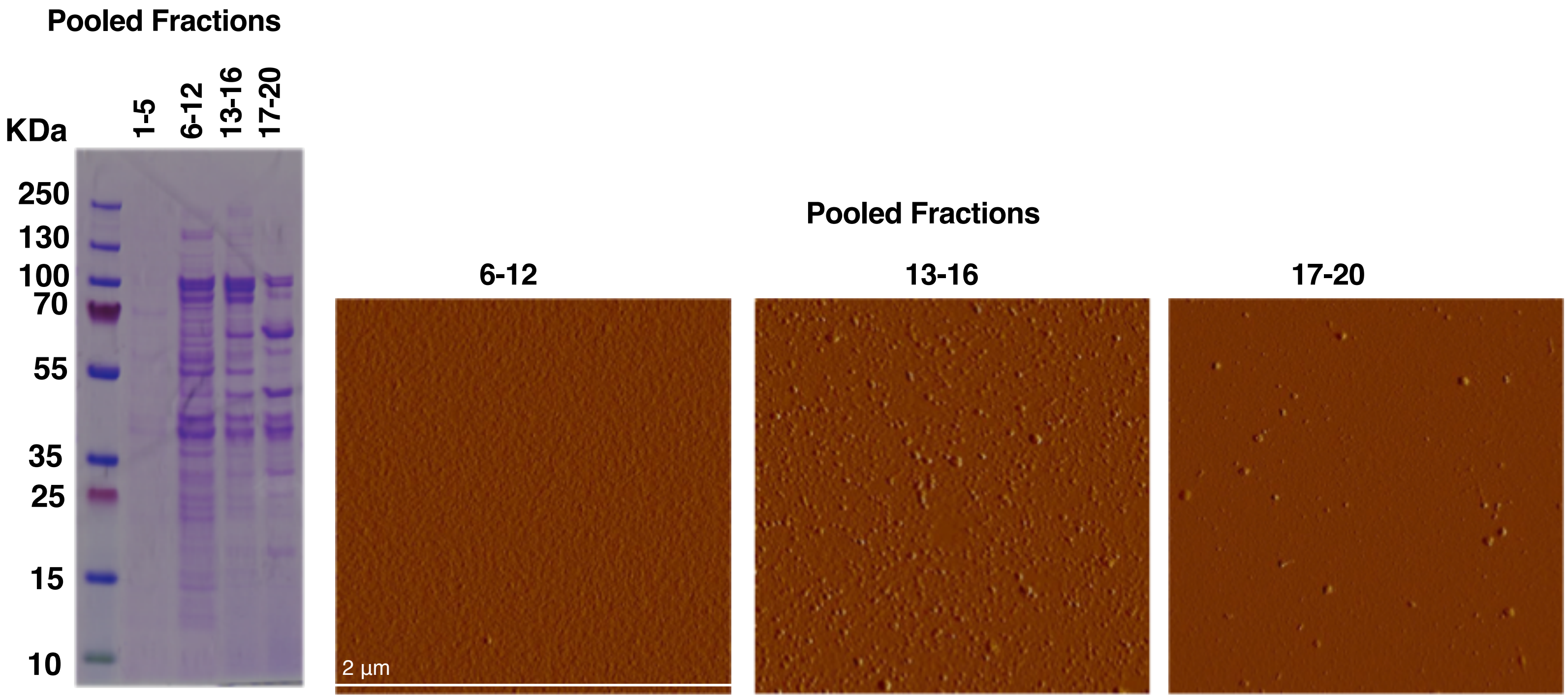

Supplementary Figure 2. Coomassie blue stained protein gel and AFM analysis of pooled fractions confirmed the purity of MV. Fractionation of MVs from *S. aureus* MSSA476 grown in bacteriologic media was carried out by density gradient centrifugation using Optiprep. 200 µl fraction aliquots were sequentially harvested and analyzed by SDS-PAGE followed by Coomassie Blue staining. The fractions showing the same protein profile were pooled (as depicted in the figure) and analyzed by AFM. The image is representative of MVs isolated from *S. aureus* MSSA476 grown in 2.5 liters BHI. The fraction number 13-16 were utilized for proteomic analysis. Scale bar is shown.

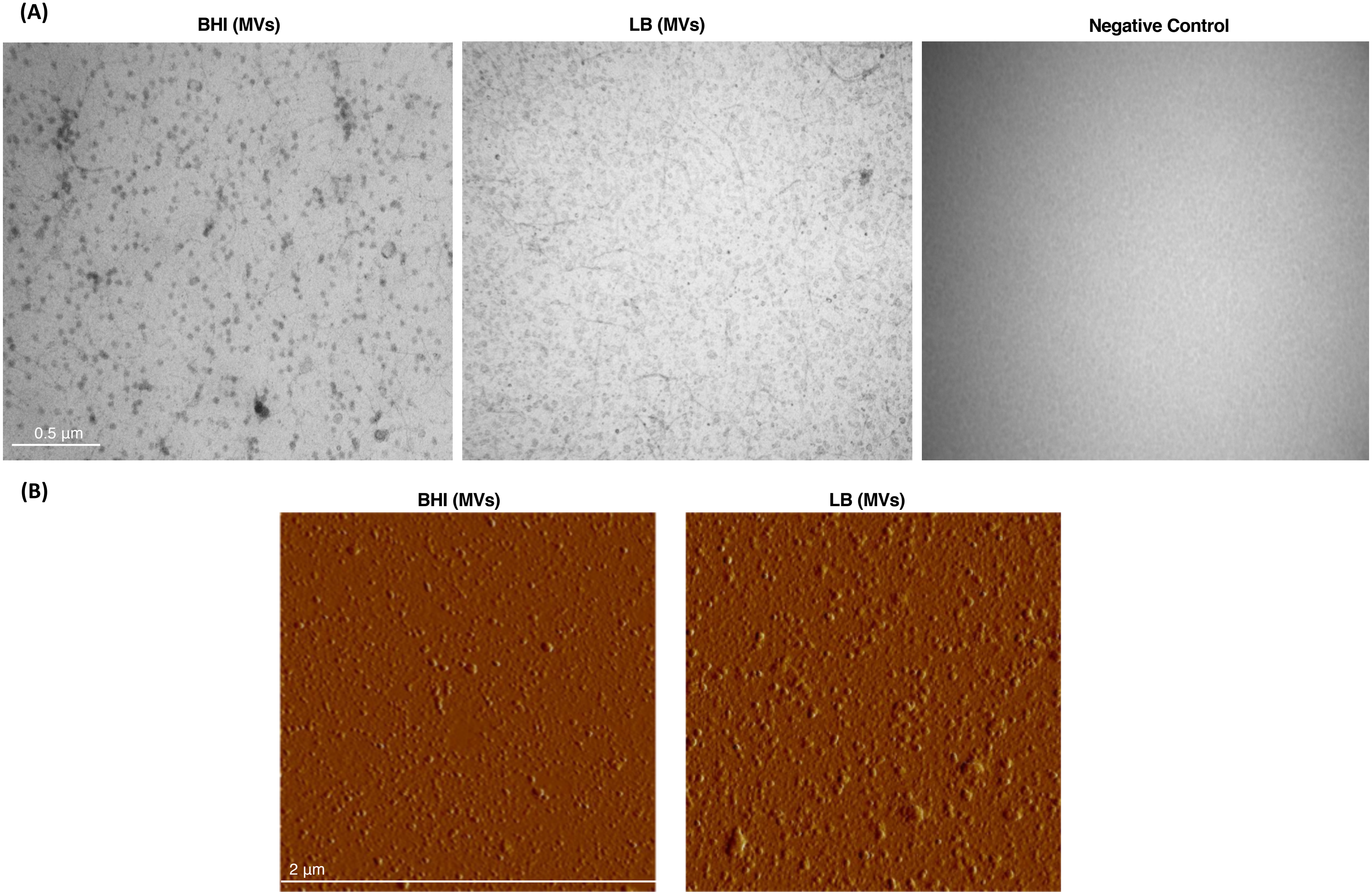

Supplementary Figure 3. *S. aureus*-derived membrane vesicles (MVs) purity were examined using TEM and AFM analysis. MVs isolated from MSSA476 grown in LB and BHI were purified by flotation through an OptiPrep density gradient. A purified MV fraction was examined using **(A)** negative staining TEM and **(B)** AFM analysis. Scale bars are shown.

Cytokine	Limit of Detection (pg/ml)
IL-β	0.52 - 8628.4
IL-2	1.02 - 19619.26
IL-4	0.29 - 5116.96
IL-5	1.26 - 21689.19
IL-17A	7.29 - 29125.51
IL-9	6.36 - 25485.92
IL-15	0.29 - 26058.25
IL-6	0.52 - 35247.83
Eotaxin	1.89 - 5997.98
IL-8	0.58 - 42703.84
MCP-1 (MCAF)	1.5 - 21590.9
RANTES	0.82 - 4239.36
INFγ	1.33 - 25394.61
TNF	3.80 - 64781.25
VEGF	1.73 - 29239.3
GM-CSF	0.74 - 13117.83

Supplementary Figure 4. The limits of detection to measure cytokine response of HaCaT cells to MVs. Level of cytokines or growth factors released from HaCaT into supernatant after 1, 3, 6 or 24 hours exposure to *S. aureus* MSSA476 MVs were analyzed using a Bio-Plex Human Cytokine 27-Plex Panel kit. The analysis was performed using high-sensitivity detection according to the manufacturer's instructions.

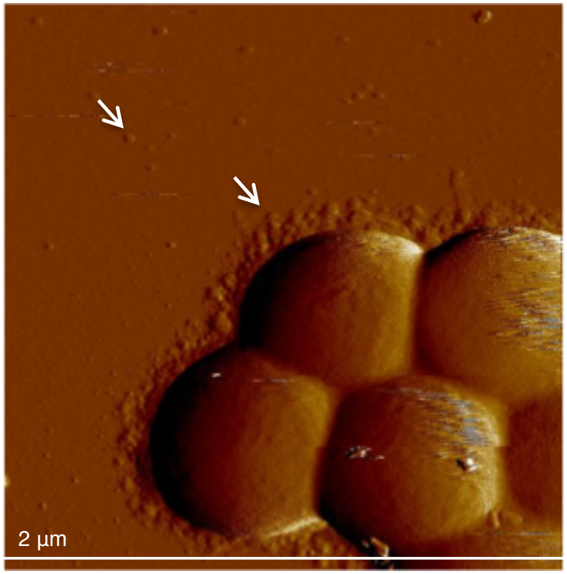

Supplementary Figure 5. MVs from *S. aureus* cultivated on LA medium. Atomic force micrograph of *S. aureus* MSSA476 cultivated on LA plate. Arrows indicate the MVs on the bacterial surface and the released MVs. Scale bars are shown.

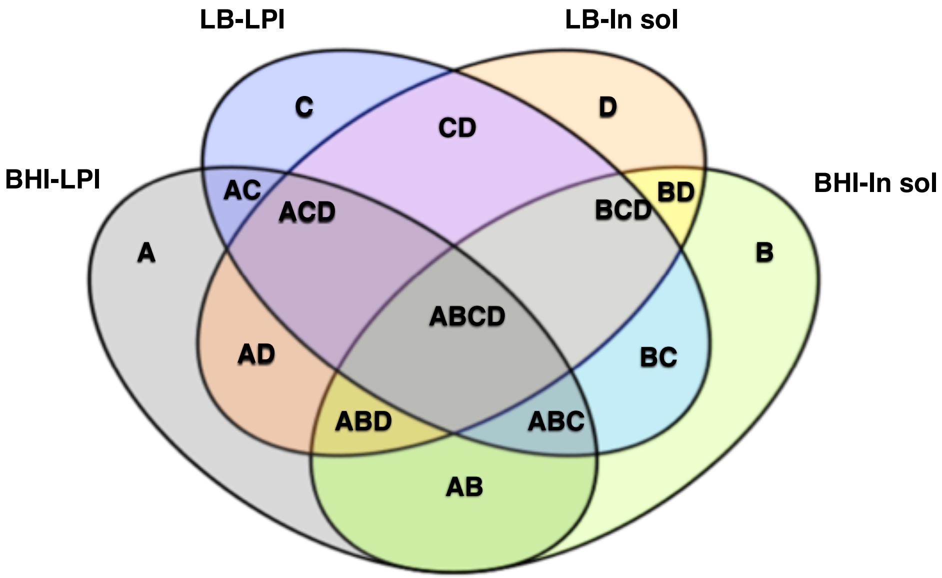

Method of Detection & Media	A	B	C	D	AB	CD	AD	BC	AC	BD	ABC	ABD	BCD	ACD	ABCD
Counts	277	36	9	7	195	6	4	0	11	0	23	7	3	15	46

Supplementary Figure 6. Comparison of *S. aureus*-MVs proteomes using LPI (A and C) and in solution (in sol, B and D) approaches. MVs were isolated from MSSA476 grown in BHI (A and B) and LB (C and D).

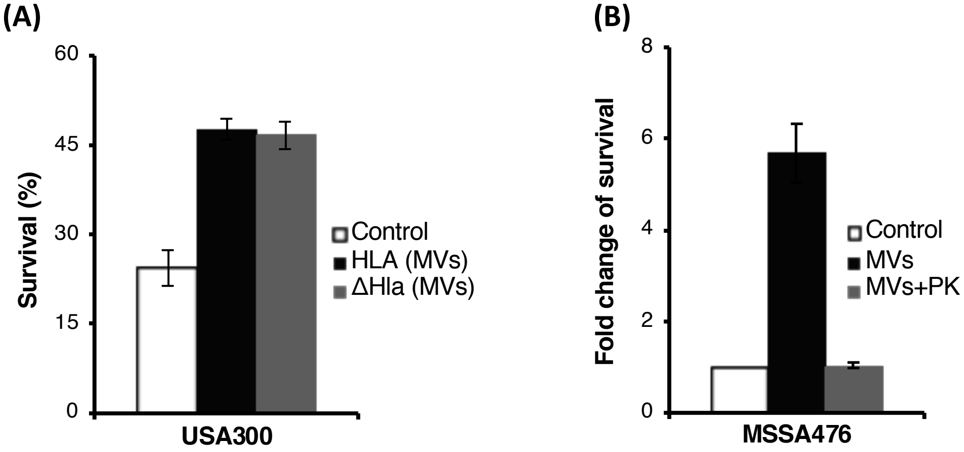

Supplementary Figure 7. *S. aureus* MVs promote bacterial survival in blood. (A) Survival of USA300 MRSA in human whole blood is increased in the presence of MVs isolated from USA300 (Hla) and USA300ΔHla (ΔHla) grown in LB. The number of inoculated bacteria at time point zero was normalized to 100% and the number of surviving bacteria after 3 hours is represented as the percentage of inoculation. **(B)** Sonication of purified MVs from MSSA476 grown in BHI followed by proteinase K (PK) treatment abolished the effect of MVs on bacterial survival in whole human blood. The number of surviving bacteria after 3 h in the absence of MVs (Control) was normalized to 1, with the number of surviving bacteria in the presence of MVs is represented as the fold change. The data are expressed as the mean ± SEM of three experiments. The significance is indicated by asterisks: ****P≤0.0001.

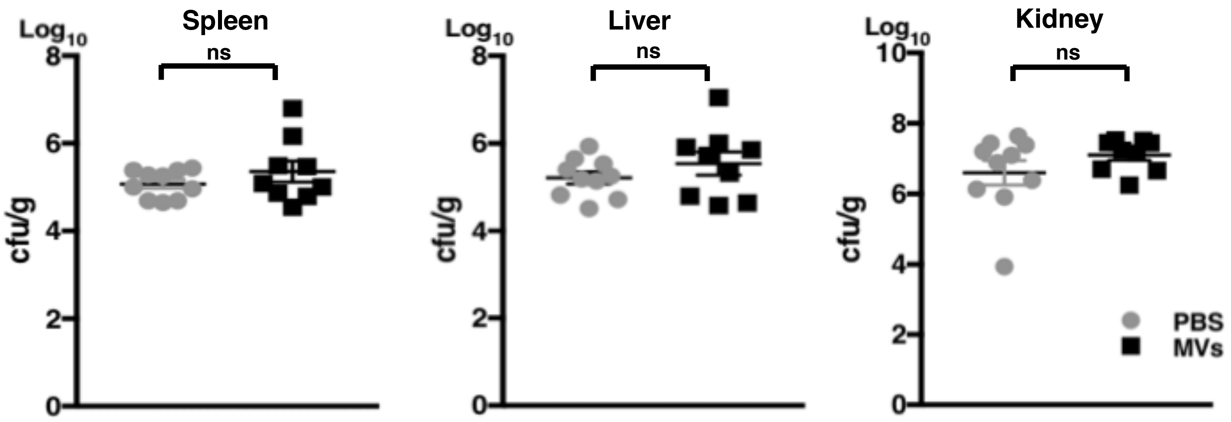

Supplementary Figure 8. ***S. aureus*** **MVs did not promote bacterial survival in different organs** ***in vivo.*** Bacterial loads in kidney, spleen, and liver (CFU/g) of eight-week-old C57BL/6 mice were counted 24 hours after the mice were intravenously infected with *S. aureus* MSSA476 supplemented with PBS or an exogenous source of MVs isolated from MSSA476 grown in BHI. Data correspond to one experiment performed with 10 mice/group (mean ± SEM). ns: no significant difference.

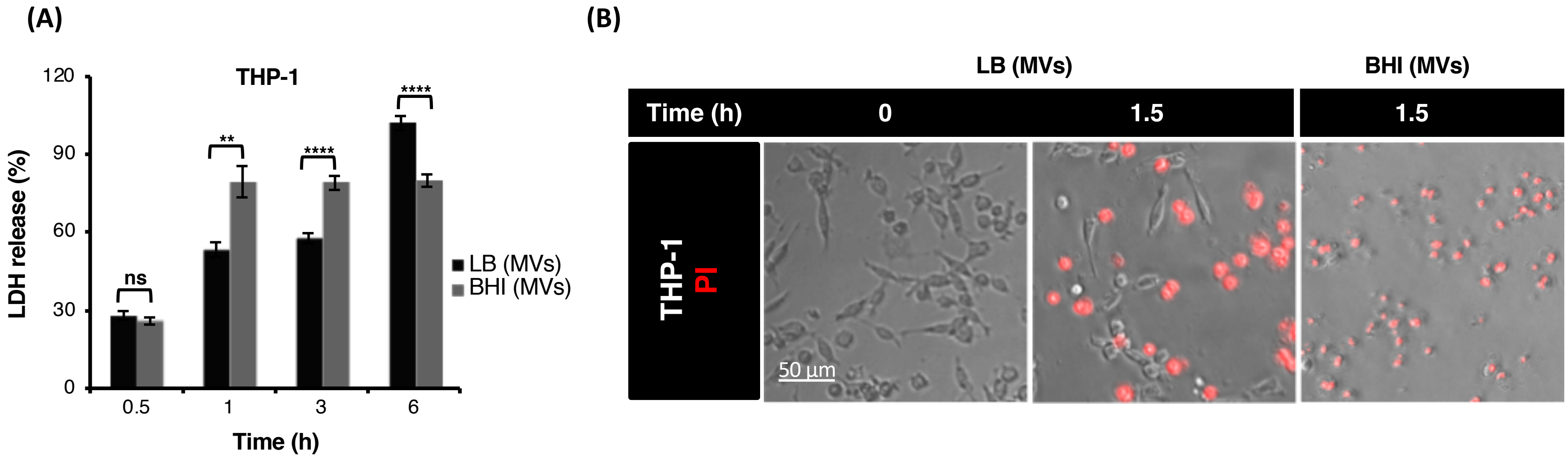

Supplementary Figure 9. *S. aureus* MVs induce cytotoxicity from macrophages. (A) THP-1 were treated with MVs (20 µg of total MVs, i.e. 0.1 µg /µl) isolated from *S. aureus* MSSA476 grown in LB or BHI at the time points indicated. Percentage of cytotoxicity was calculated by measuring the amount of LDH released from the cytosol of damaged cells into the supernatant after exposure to MVs. The data are expressed as the mean ± SEM of three independent experiments. The Significance is indicated by asterisks: ***P*≤0.01; *****P*≤0.0001. ns: no significant difference. **(B)** Viability staining of THP-1 cells was performed using propidium iodide (PI). Live imaging was performed after 0 and 0.45 or 1.5 h using fluorescence microscopy. Scale bar is shown.

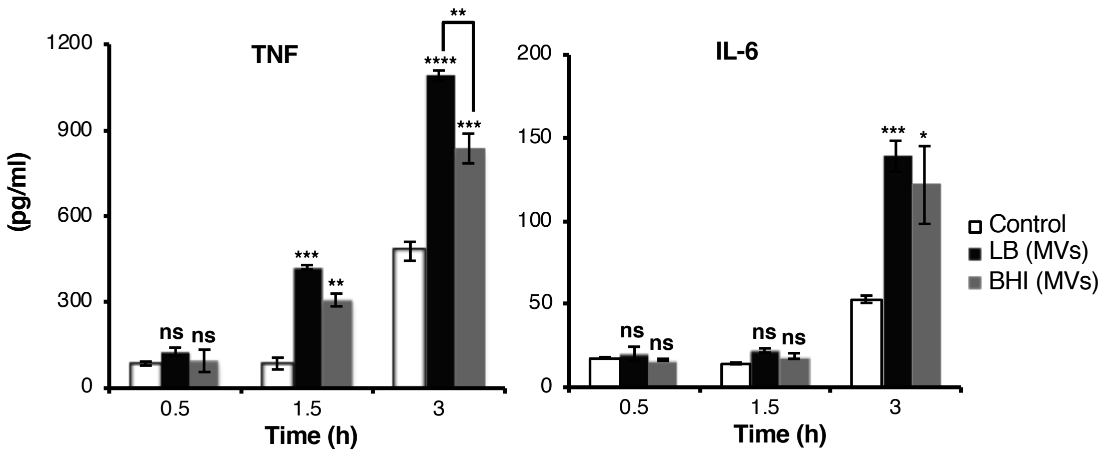

Supplementary Figure 10. *S. aureus* MVs elicit the release of pro-inflammatory cytokines from macrophages. Level of cytokines released from THP-1 cells into supernatant after 0.5, 1.5 and 3 h exposure to *S. aureus* MSSA476 MVs isolated from bacteria grown in LB or BHI. The data are expressed as the mean ± SEM of three independent experiments. The significance is indicated by asterisks: *P<0.05; **P≤0.01. ***P≤0.001; ****P≤0.0001. ns: no significant difference.

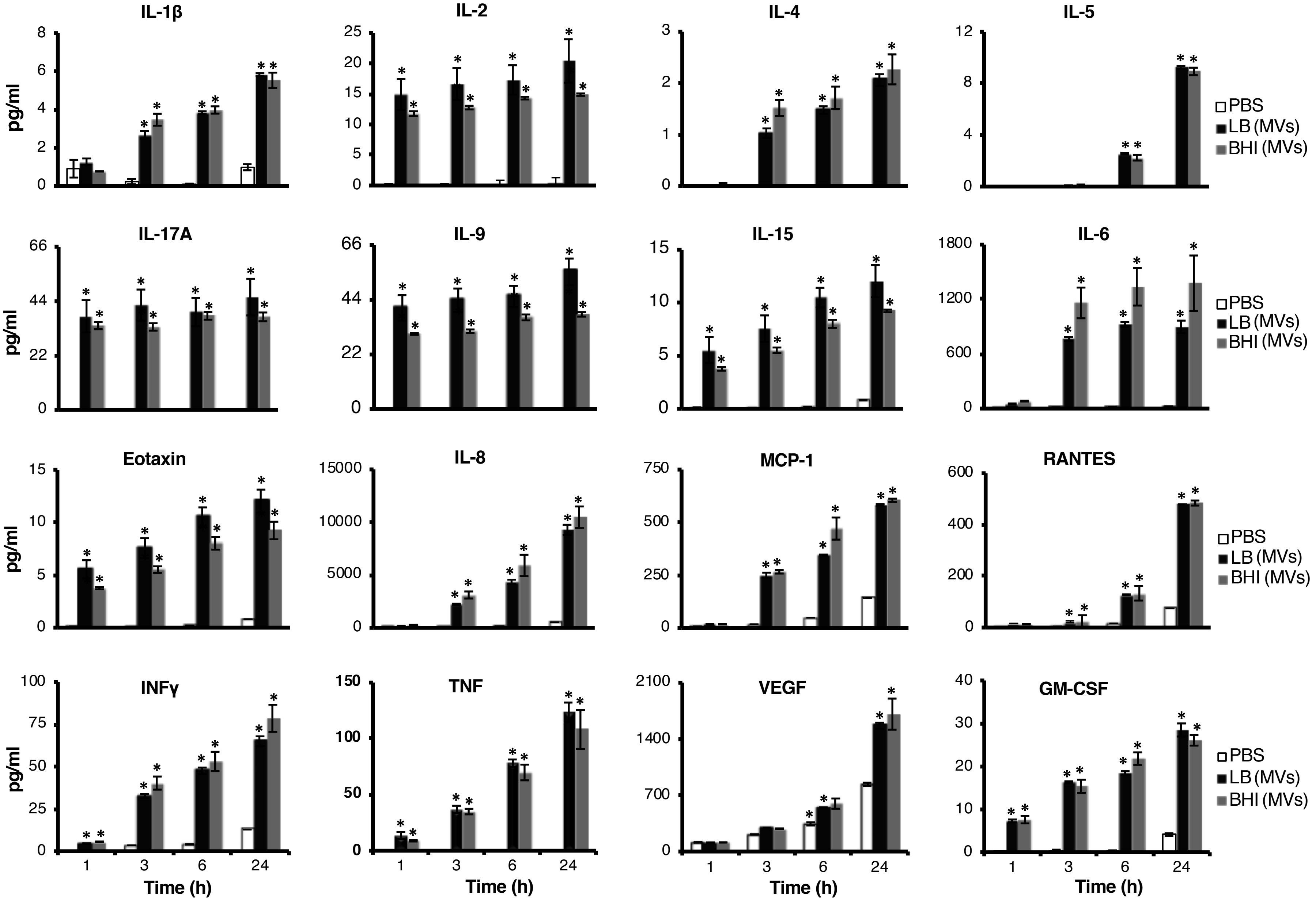

Supplementary Figure 11. ***S. aureus* MVs elicit the release of cytokines in human cells.** Level of cytokines or growth factors released from HaCaT into supernatant after 1, 3, 6 or 24 h exposure to *S. aureus* MSSA476 MVs isolated from bacteria grown in LB or BHI. The data are expressed as the mean ± SEM of three independent experiments. The significance is indicated by asterisk: *P<0.05.
